# CDX2-induced intestinal metaplasia in human gastric organoids derived from induced pluripotent stem cells

**DOI:** 10.1016/j.isci.2022.104314

**Published:** 2022-04-28

**Authors:** Takahiro Koide, Michiyo Koyanagi-Aoi, Keiichiro Uehara, Yoshihiro Kakeji, Takashi Aoi

**Affiliations:** 1 Division of Advanced Medical Science, Graduate School of Science, Technology and Innovation, Kobe University, Kobe, Japan; 2 Department of iPS Cell Applications, Graduate School of Medicine, Kobe University, Kobe, Japan; 3 Division of Gastrointestinal Surgery, Department of Surgery, Graduate School of Medicine, Kobe University, Kobe, Japan; 4 Center for Human Resource Development for Regenerative Medicine, Kobe University Hospital, Kobe, Japan; 5 Department of Diagnostic Pathology, Graduate School of Medicine, Kobe University, Kobe, Japan

**Keywords:** Biological sciences, Cell biology, Stem cells research

## Abstract

Intestinal metaplasia is related to gastric carcinogenesis. Previous studies have suggested the important role of CDX2 in intestinal metaplasia, and several reports have shown that the overexpression of CDX2 in mouse gastric mucosa caused intestinal metaplasia. However, no study has examined the induction of intestinal metaplasia using human gastric mucosa. In the present study, to produce an intestinal metaplasia model in human gastric mucosa *in vitro*, we differentiated human-induced pluripotent stem cells (hiPSC) to gastric organoids, followed by the overexpression of CDX2 using a tet-on system. The overexpression of CDX2 induced, although not completely, intestinal phenotypes and the enhanced expression of many, but not all, intestinal genes and previously reported intestinal metaplasia-related genes in the gastric organoids. This model can help clarify the mechanisms underlying intestinal metaplasia and carcinogenesis in human gastric mucosa and develop therapies to restitute precursor conditions of gastric cancer to normal mucosa.

## Highlights

• We established the drug-inducible CDX2 expression in gastric organoids from hiPSCs
• Intestinal phenotypes were induced, although not completely, in the gastric organoids
• Many, but not all, intestinal genes and metaplasia-related genes were upregulated
• Endogenous CDX2—an intestinal master transcription factor—was not upregulated

## Graphical abstract

**Figure:**
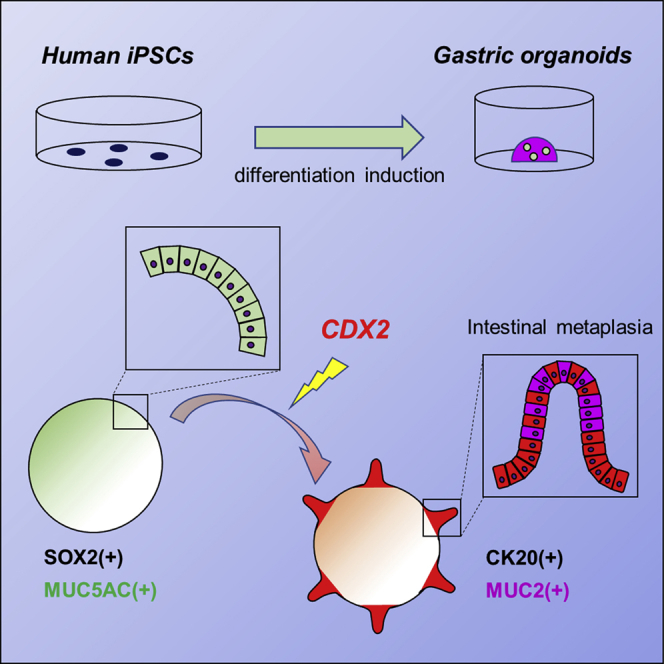

## Introduction

Gastric intestinal metaplasia frequently accompanies intestinal-type gastric cancer (Correa, 1992; Correa and Shiao, 1994; Plummer et al., 2015). Intestinal metaplasia has been regarded as a precancerous lesion in the *Helicobacter pylori*-induced metaplasia-dysplasia-carcinoma sequence (de Vries et al., 2008). However, some researchers have argued that intestinal metaplasia is a para-cancerous lesion, because more than two-thirds of microscopic gastric cancer do not have the intestinal phenotype (Kawachi et al., 2003) and because of inconsistencies in the phenotype expression of mucin between gastric cancers and the surrounding mucosa (Hattori, 1986). Regardless of whether gastric intestinal metaplasia is a precancerous or para-cancerous lesion, understanding the molecular mechanisms of intestinal metaplasia is important for clarifying the details of gastric carcinogenesis.

CDX2—an intestinal specific homeobox gene—has been suggested to play a crucial role in intestinal metaplasia as well as in the development and maintenance of the intestinal mucosa phenotype (Beck et al., 1999; Chen et al., 2021). The expression of CDX2 as well as CDX1 is confined to the posterior gut endoderm during later development and after birth (Guo et al., 2004). Cdx2 heterozygous knockout mice develop multiple intestinal polyp-like lesions that do not express Cdx2 and contain areas of squamous metaplasia (Grainger et al., 2013). Indeed, CDX2 and CDX1 are expressed in human gastric epithelial metaplasia (Almeida et al., 2003).

Furthermore, several groups have shown that transgenic expression of CDX2 in mouse gastric mucosa results in intestinal metaplasia (Mutoh et al., 2002; Silberg et al., 2002). However, no intestinal metaplasia model of human gastric mucosa has been reported because transgenic experiments in humans are impossible. To resolve this issue, a range of human pluripotent stem cell differentiation technologies have been developed over the past decade, thereby enabling the creation of various types of human tissues *in vitro—*also referred to as “organoids”—including gastric mucosa, which can be used in transgenic experiments (McCracken et al., 2014, 2017).

In this study, we tried to establish a human gastric intestinal metaplasia model by overexpressing CDX2 in gastric organoids derived from pluripotent stem cells and achieved incomplete metaplasia. This model can help clarify the mechanisms of intestinal metaplasia and carcinogenesis in human gastric mucosa and develop therapies to restitute precursor conditions of gastric cancer to normal mucosa.

## Results

### Validation of a human-induced pluripotent stem cell (hiPSC) line to differentiate into gastric organoids

In this study, we used a hiPSC line FF-PB-3AB4 generated from a healthy donor’s peripheral blood mononuclear cells (PBMCs) using episomal plasmid vectors under xeno-free and feeder-free conditions (Suzuki et al., 2019). FF-PB-3AB4 showed hES cell-like morphologies, just like the reference iPSC line 201B7 that was cultured under the same conditions (Figure S1A), and expressed the pluripotent markers OCT3/4, SOX2, and NANOG at the mRNA (Figure S1B) and protein levels (Figure S1C). FF-PB-3AB4 successfully differentiated into three germ layers (Figure S1D) *in vitro* and had a normal karyotype (Figure S1E).

Next, we examined whether or not this iPSC line could differentiate into three-dimensional antral gastric organoids using a previously reported *in vitro* culture system (McCracken et al., 2014, 2017). Phase contrast microscopy showed sequential morphological changes from undifferentiated iPSC to definitive endoderm (DE), foregut, and gastric organoids (Figure S2A). A quantitative reverse transcription polymerase chain reaction (qRT-PCR) analysis showed that the endoderm markers SOX17 and FOXA2 were upregulated at the early stages; however, the foregut marker—SOX2—was continuously expressed but downregulated in later stages (Figure S2B). The gastric pyloric epithelial cell marker—PDX1—and gastric surface mucous cell marker—MUC5AC—were upregulated at the late stages (Figure S2B). The morphology of gastric organoids at day 61 is shown in Figure S3A. On HE staining, gastric epithelial cellular composition such as foveolar cells, parietal cells, chief cells, and neck mucous cells was unclear (Figure S3B top panels). However, immunostaining clearly showed that most cells were positive for MUC5AC—a gastric foveolar cell marker (Figures S3B and S4). Furthermore, we observed a few cells positive for H,K-ATPase (ATP4A) (Figure S5A)—a parietal cell marker—and for the endocrine cell markers—Somatostatin (SST), Synaptophysin (SYP), and Chromogranin A (CHGA) (Figure S5B). We identified cells with obvious expression of SOX2 and PDX1 but no expression of the intestinal markers CDX2 or MUC2 on an immunohistological analysis (Figures S3B and S4).

### Generation of hiPSC with drug-inducible CDX2 expression

**Figure 1:**
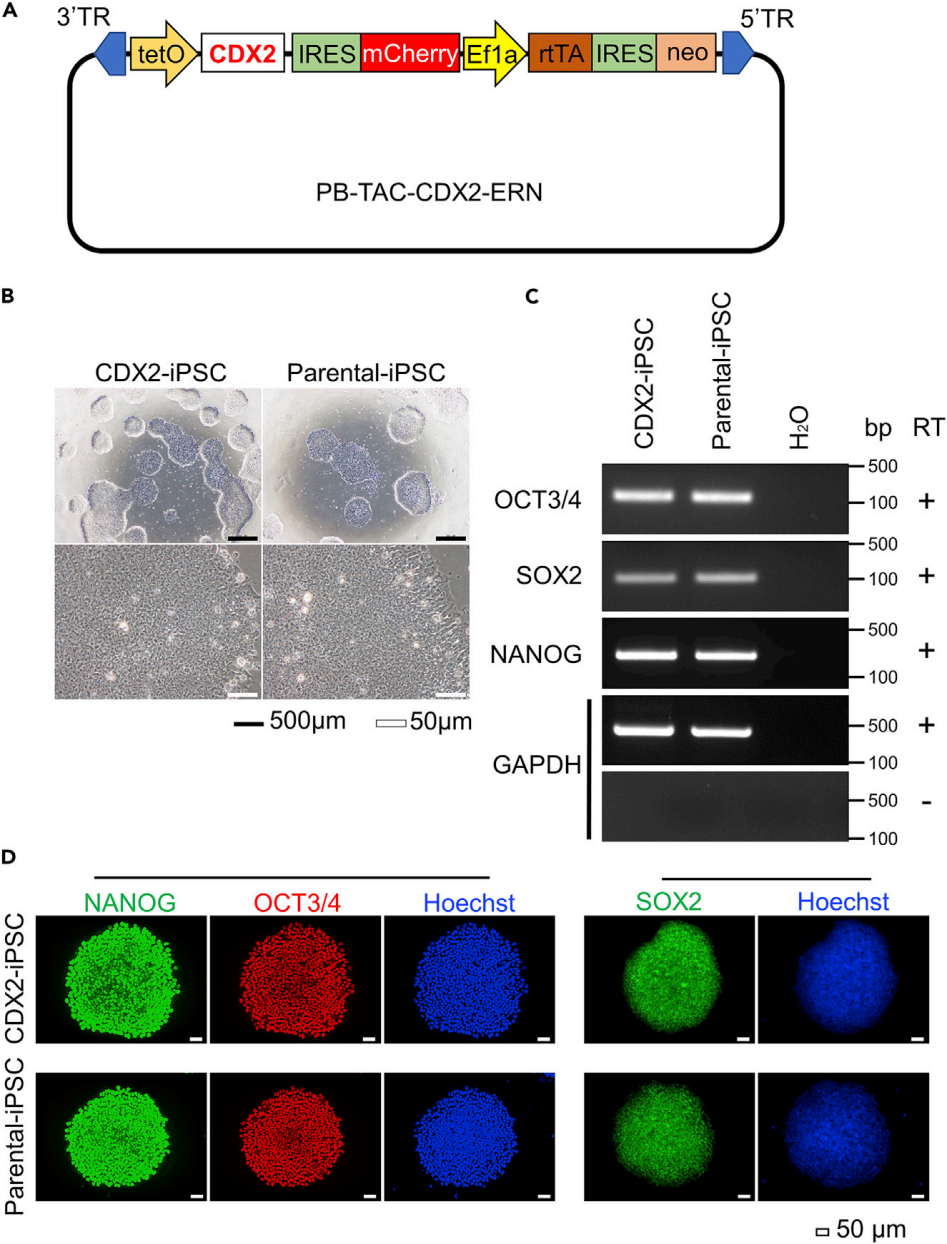
The production of the PB-TAC-CDX2-ERN vector and introduction into iPSC (A) A schematic diagram of the piggyBac vector containing doxycycline (DOX)-inducible CDX2 (PB-TAC-CDX2-ERN). (B) The cell morphologies of CDX2-iPSC (left panels) and iPSC before transfection of the PB-TAC-CDX2-ERN vector: Parental-iPSC (right panels). Scale bars: black bars = 500 μm, white bars = 50 μm. (C) An RT-PCR analysis showed that CDX2-iPSC maintained the mRNA expression of the pluripotent markers OCT3/4, SOX2, and NANOG. GAPDH was used as an endogenous control. RT: reverse transcriptase. (D) Immunostaining showed that CDX2-iPSC (upper panels) as well as Parental-iPSC (lower panels) expressed the pluripotency markers NANOG, OCT3/4, and SOX2. The nuclei were stained blue with Hoechst33342. Scale bars, 50 μm.

To induce the forced expression of CDX2 in the gastric organoids from hiPSC, we constructed the DOX-inducible PB-transposon plasmid PB-TAC-CDX2-ERN (Figure 1A) and introduced it with pCAG-PBase into the parental iPSC line FF-PB-3AB4. We then isolated the subclone that expressed mCherry after DOX treatment, subsequently referred to as “CDX2-iPSC.” Similar to the parental iPSC, CDX2-iPSC showed hES cell-like morphologies (Figure 1B) and expressed the undifferentiated markers OCT3/4, SOX2, and NANOG at the mRNA (Figure 1C) and protein levels (Figure 1D).

**Figure 2:**
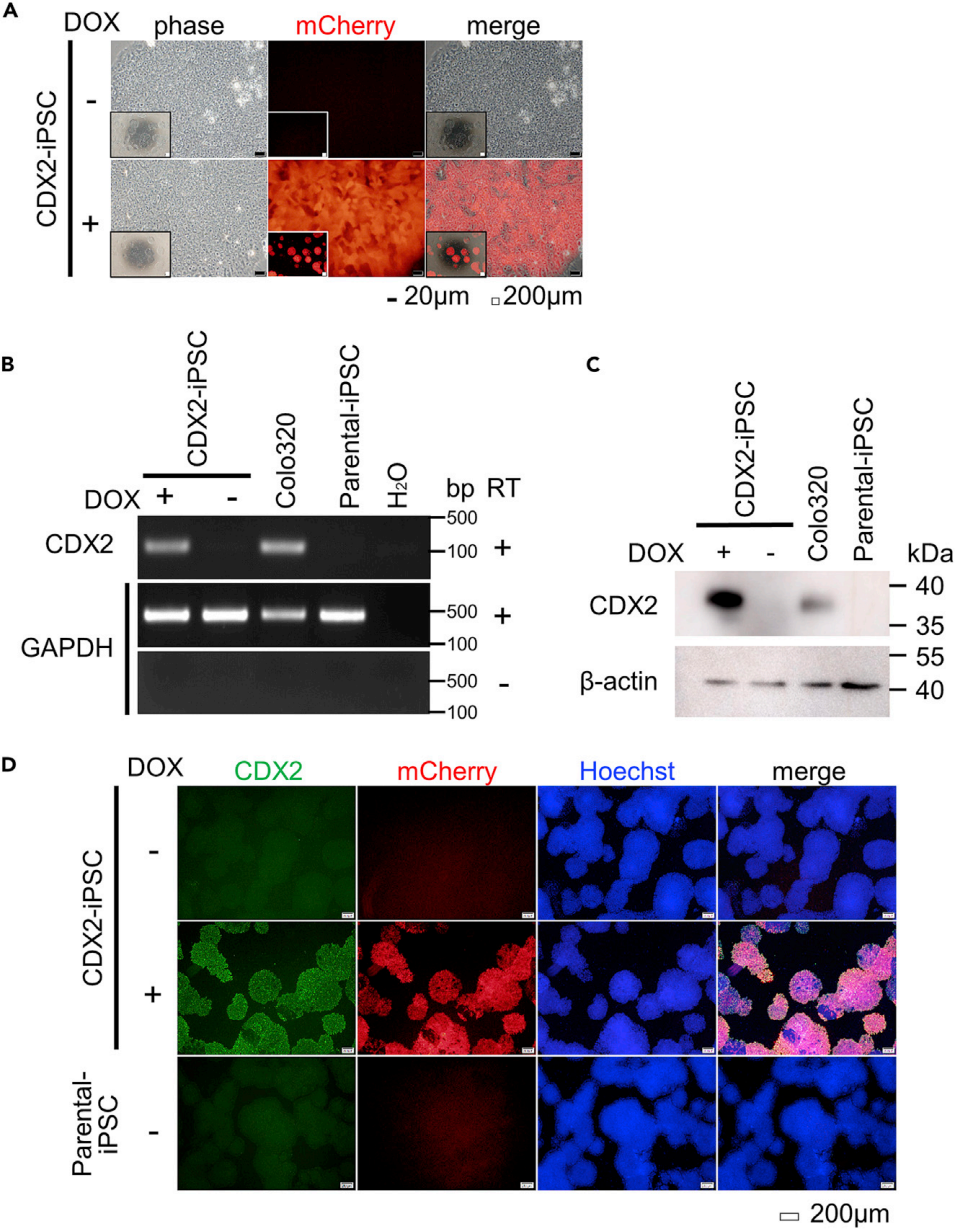
DOX-inducible CDX2 in CDX2-iPSC (A) A fluorescence analysis of the mCherry expression in CDX2-iPSC with (lower panels) or without (upper panels) DOX administration for 24 h. The representative data of three independent experiments are shown. Scale bars: black bars = 20 μm, white bars = 200 μm. (B) An RT-PCR analysis of the total CDX2 expression in CDX2-iPSC. The colorectal cancer cell line Colo320 was used as a positive control of the CDX2 expression. GAPDH was used as an endogenous control. CDX2-iPSC in culture with DOX administration showed CDX2 expression. Representative data of two independent experiments are shown. (C) Western blotting showing the protein expressions of CDX2 and β-actin in CDX2-iPSC cultured with or without DOX administration as well as Colo320. Cell lysates were collected at 2 days post-tet-on. Representative data of three independent experiments are shown. (D) Immunostaining of CDX2 in CDX2-iPSC at 2 days post-DOX administration. CDX2 was expressed in CDX2-iPSC cultured with DOX and co-localized with mCherry. Scale bars, 200 μm.

We confirmed that almost all of the CDX2-iPSC expressed mCherry by adding 1 μM of Doxycycline (DOX) for 24 h (Figure 2A). The upregulation of CDX2 mRNA and protein was detected by RT-PCR (Figure 2B) and Western blotting (Figure 2C), respectively. Immunostaining showed that CDX2 was co-expressed with mCherry in CDX2-iPSC with DOX but not in parental iPSC or CDX2-iPSC without DOX (Figure 2D).

### Drug-inducible CDX2 expression in gastric organoids from CDX2-iPSC

**Figure 3:**
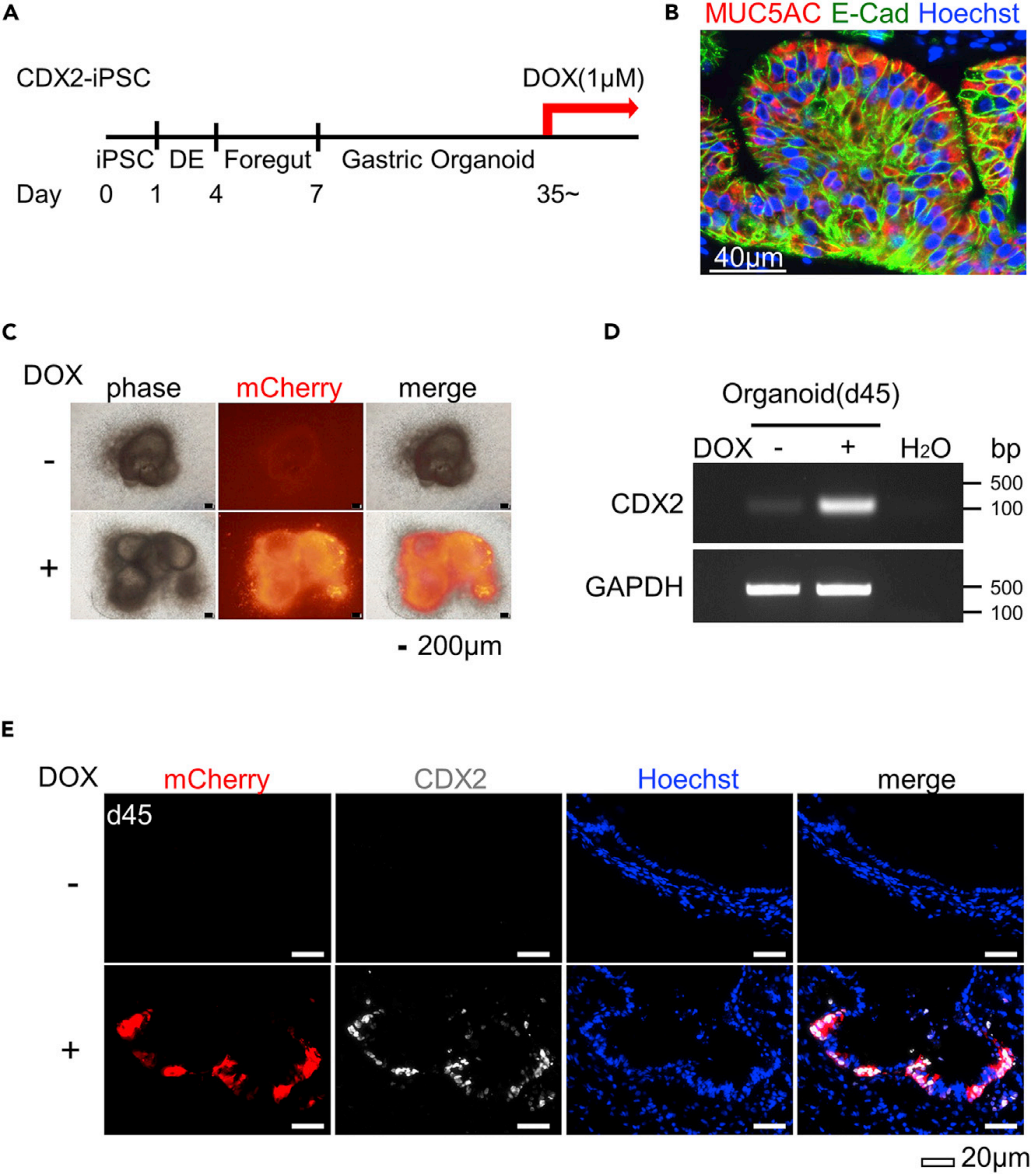
The overexpression of CDX2 in induced gastric organoids (A) A schematic representation of the protocol of gastric organoid differentiation from CDX2-iPSC. The administration of DOX (1 μM) started between days 35 and 40. (B) Immunofluorescence analyses of the MUC5AC and E-cadherin (E-Cad) expression in differentiated gastric organoids at day 37 without DOX treatment. Scale bar, 40 μm. (C) Morphologies of gastric organoids derived from CDX2-iPSC after 1 μM DOX treatment for 10 days from Day 36. The representative data of three independent experiments are shown. Scale bars, 200 μm. (D) An RT-PCR analysis showed the mRNA expression of CDX2 at Day45 with or without DOX treatment for 9 days. The expression of CDX2 was increased in DOX(+) organoids. GAPDH was used as an endogenous control. (E) Immunofluorescence analyses of CDX2 and mCherry in gastric organoids derived from CDX2-iPSC at Day45 with or without DOX treatment for 10 days. Scale bars, 20 μm.

Next, we established a system for drug-inducible forced expression of CDX2 in gastric epithelium derived from hiPSC (Figure 3A). Consistent with gastric differentiation from the parental iPSC line, the differentiation progeny of CDX2-iPSC formed gastric organoids and expressed the gastric epithelial marker MUC5AC (Figures 3B and S6A). Because the expression of MUC5AC was upregulated at approximately 30 days in the parental iPSC-derived organoids (Figure S2B), we decided to start adding DOX after day 35.

We then tested whether or not DOX treatment upregulates the expression of CDX2 in the hiPSC-derived gastric organoids. The fluorescence of mCherry protein (Figure 3C) and the enhanced expression of CDX2 mRNA (Figure 3D) were detected in gastric organoids treated with 1 μM of DOX for 9 days but not in organoids without DOX. Furthermore, immunohistochemistry showed that CDX2 was co-expressed with mCherry protein by adding DOX (Figures 3E and S6B). Taken together, these findings indicate that we successfully induced the forced expression of CDX2 upon DOX addition in hiPSC-derived gastric organoids.

### Intestinal metaplasia in gastric organoids

**Figure 4:**
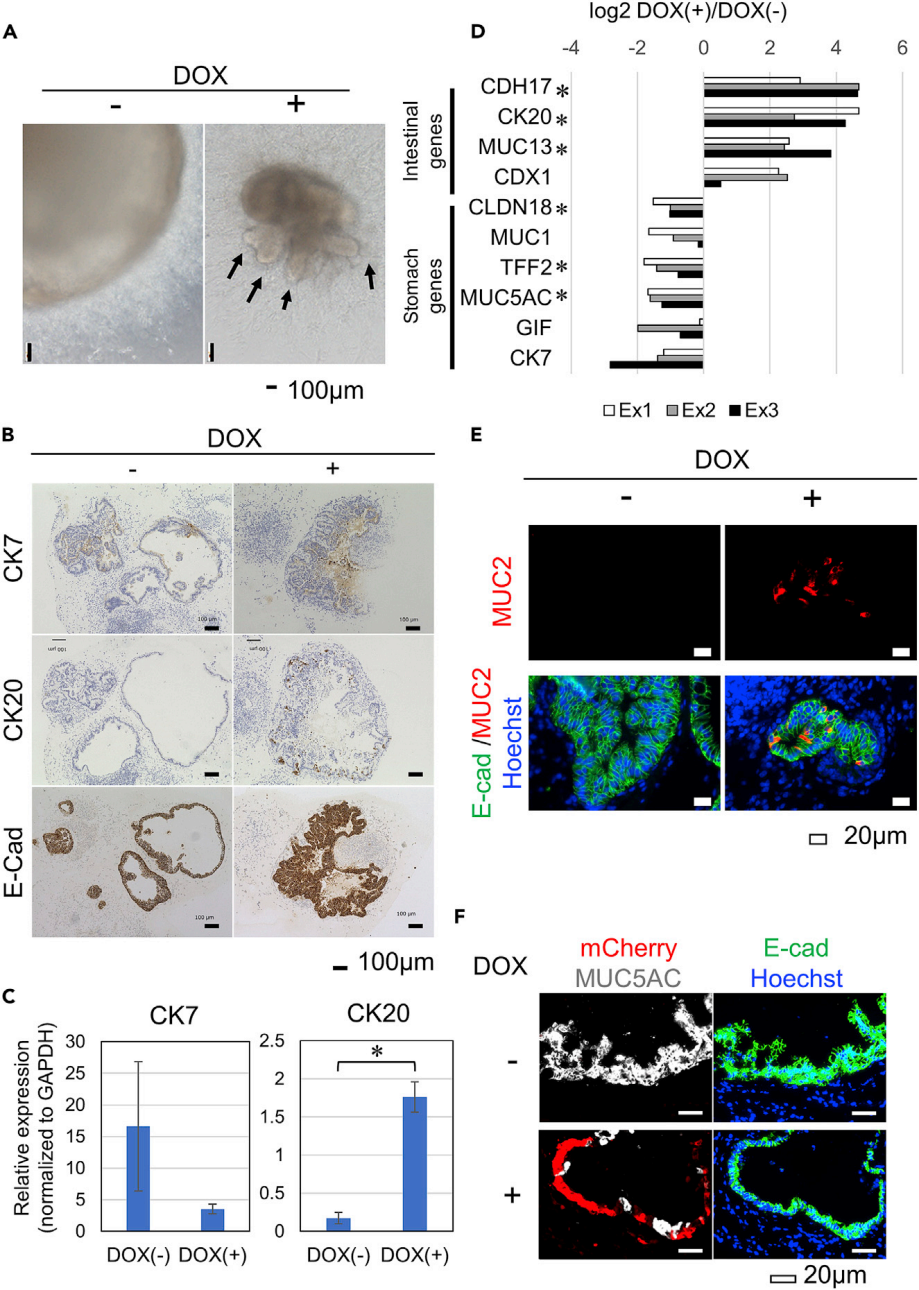
Phenotype alterations of DOX(+) organoids (A) Phase contrast micrographs of the morphologies of DOX(−) organoids (Day 42, left panel) and DOX(+) organoids (Day 42, right panel). Arrows indicate crypt-like structures. Scale bars, 100 μm. (B) Immunohistochemical analyses of CK7, CK20, and E-Cadherin in the gastric organoids derived from CDX2-iPSC at day 40 with (+) or without (−) DOX treatment for 5 days. Scale bars, 100 μm. (C) qRT-PCR analysis showed the mRNA expressions of CK7 and CK20. The expression of CK20 was increased in DOX(+) organoids. The mRNA expression was normalized to GAPDH. Data are represented as mean ± SEM of three independent induction experiments. ∗, p < 0.05. (D) The difference in the expression of the intestinal markers and the stomach markers between DOX(+) and DOX(−) organoids was calculated by subtracting the log2 RPKM value of DOX(−) from that of DOX(+). ∗, p < 0.05. (E) Immunofluorescence analyses of E-Cadherin and MUC2 expression in the gastric organoids derived from CDX2-iPSC at Day 40 with (+) or without (−) DOX treatment for 5 days. Scale bars, 20 μm. (F) Immunofluorescence analyses of E-Cadherin and MUC5AC in gastric organoids from CDX2-iPSC at Day 45 with (+) (lower panels) or without (−) (upper panels) DOX treatment for 10 days. Scale bars, 20 μm.

When we treated the iPSC-derived organoids with DOX, phase contrast microscopy revealed the budding of crypt-like domains (Figures 4A and S7A), which have been found in mouse and human intestinal organoids, but not gastric organoids derived from *in vivo* tissues (Barker et al., 2010; Fordham et al., 2013; Jung et al., 2011; Sato et al., 2009).

We evaluated the expression patterns of CK7 and CK20 in the epithelial cells in the organoids. The cells facing the lumen were considered to be epithelial cells, as all such cells were positive for E-cadherin (Figures 4B and S8A lower panels). The epithelium in DOX(−) organoids contained CK7-positive and CK7-negative cells (Figures 4B and S8A upper left panels), which was compatible with the fact that fetal gastric epithelium at a certain point in time contains simultaneously CK7-positive and CK7-negative cells (Kirchner et al., 2001). Notably, in contrast to all epithelial cells in the DOX(−) organoids being negative for CK20, CK20-positive epithelial cells appeared in DOX(+) organoids, which contained CK7-positive and CK7-negative cells (Figures 4B and S8A upper and middle right panels). A quantitative immunohistochemical analysis of three independent experiments revealed a statistically significant emergence of CK20-positive cells in DOX (+) organoids (Figure S8B). The immunophenotype of gastric intestinal metaplasia has been known to be CK7(−)/CK20(+) or CK7(+)/CK20(+) (Ormsby et al., 1999; Couvelard et al., 2001), and our results suggested that intestinal metaplasia could be induced in the DOX(+) organoids (Figures 4B, S6C, and S8A). The enhanced expression of CK20 in DOX(+) organoids was also confirmed with qRT-PCR (Figure 4C). An RNA-seq analysis showed that the forced expression of CDX2 resulted in the statistically significant upregulation of CK20 as well as other intestinal markers (CDH17, MUC13) and a tendency toward the upregulation of another intestinal marker, CDX1. In addition, the statistically significant downregulation of some stomach markers (CLDN18, TFF2, and MUC5AC) and a tendency toward the downregulation of other stomach markers (MUC1, GIF, and CK7) (Figure 4D) were also noted. Furthermore, we detected cells expressing the intestinal marker MUC2 in DOX(+) organoids but not in DOX(−) organoids in immunofluorescence analyses in all four independent induction experiments (Figures 4E, S6D, and S7B). MUC2-positive cells were clustered in the crypt-like area of the DOX(+) organoid (Figure S7B). In contrast, the DOX(−) organoids showed neither such a structure nor any MUC2-positive cells. These results revealed a significant emergence of MUC2-positive cells in DOX(+) organoids (chi-square test, p < 0.05).

Cells expressing E-cad, which is an indicator of epithelial cells, in DOX(−) organoids expressed MUC5AC, indicating a gastric epithelial phenotype (Figure 4F upper panels and Figure S6E). In contrast, mCherry (+) epithelial cells did not express MUC5AC, although all mCherry (−) epithelial cells expressed MUC5AC in DOX(+) organoids (Figure 4F lower panels). A quantitative immunofluorescence analysis of three independent experiments revealed that the expression of MUC5AC was significantly disappeared in mCherry-positive cells, i.e., CDX2-overexpressing cells of DOX(+) organoids (Figure S8C).

### Effect of CDX2 forced expression on the genome-wide gene expression profile of hiPSC-derived gastric organoids

We compared the genome-wide gene expression patterns between hiPSC-derived gastric organoids with and without the forced expression of the CDX2 gene using RNA-seq analysis. To assess the induction of the intestinal gene expression pattern, we employed two gene lists: a list consisting of genes highly expressed in the normal intestine compared to the normal stomach (“Intestinal Genes”) and a publicly available list of intestinal metaplasia marker genes (“IM Genes”) obtained by a microarray analysis of microdissected human intestinal metaplasia tissues (Lee et al., 2010). To create the first list, we analyzed the previously reported transcriptome data of human tissues (Fagerberg et al., 2014) (see the STAR Methods section).

To confirm the reliability of our experiments, we first performed a clustering analysis in “All Genes,” “Intestinal Genes,” and “IM Genes.” According to this analysis, the six samples of the organoids generated among the three independent experiments could be clearly divided into two groups: one consisting of only DOX(−) samples and the other consisting of only DOX(+) samples, indicating the reproducibility of our experimental system (Figure S9).

**Figure 5:**
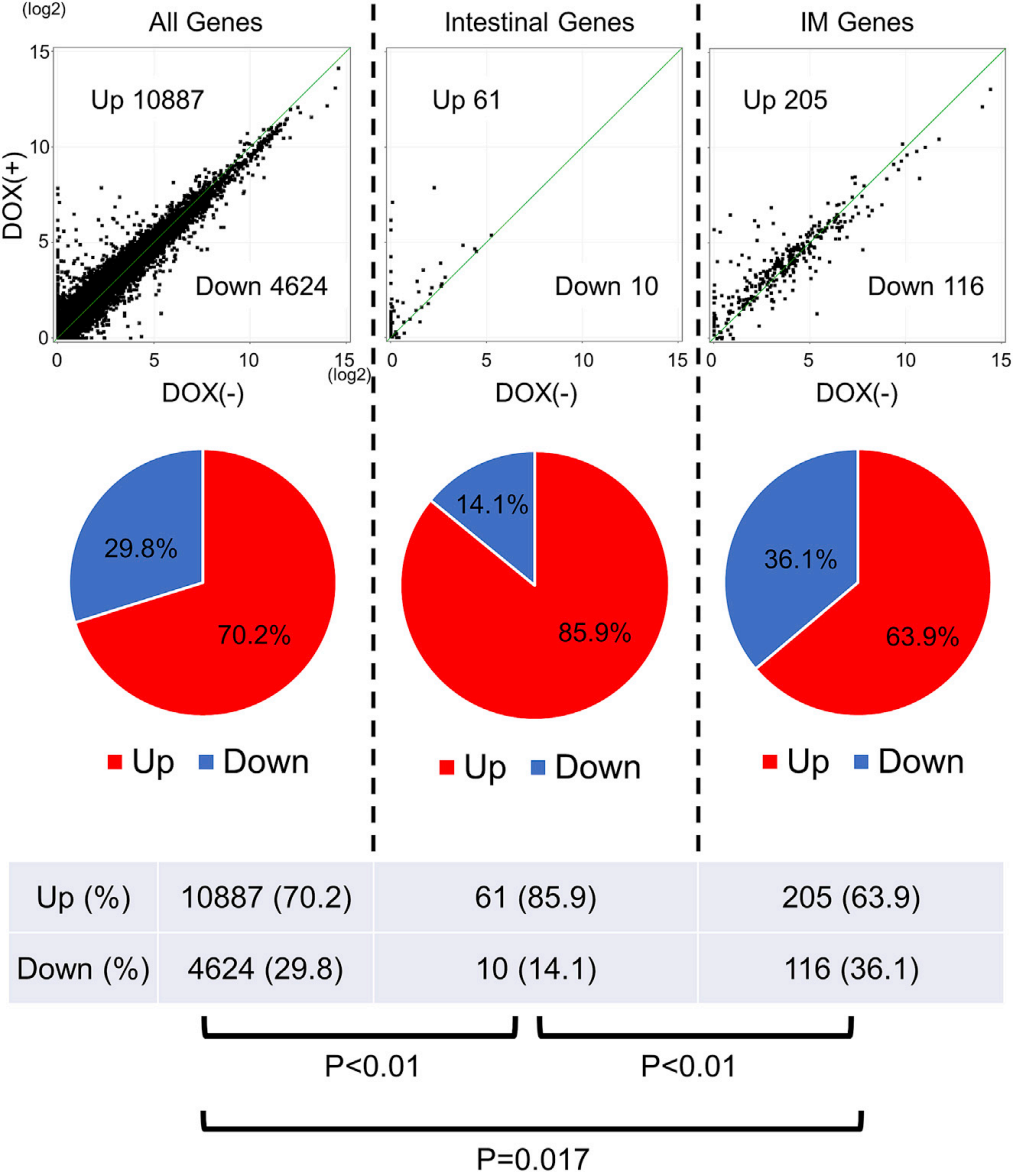
Analyses of the global gene expression changes in CDX2-overexpressing gastric organoids Scatter plot analyses of the all genes (left panel), highly expressed genes in the normal intestine compared to the normal stomach (“Intestinal Genes,” middle panel) and gene for intestinal metaplasia marker genes (Lee et al., 2010) (“IM Genes,” right panel) in DOX(+) gastric organoids (y axis, n = 3) versus DOX(−) gastric organoids (x axis, n = 3) after the addition of DOX for 7 days. Pie charts (middle panels) and tables (lower panels) show the percentage of genes that showed an increased or a decreased expression.

RNA-seq analysis for all genes showed that 10887 (70.2%) and 4624 genes (29.8%) were upregulated and downregulated in the DOX(+) organoids, respectively (Figure 5 upper left panel). When we focused on the “Intestinal Genes” group, 61 genes (85.9%) were upregulated, whereas only 10 (14.1%) were downregulated in the DOX(+) organoids (Figure 5 upper middle panel). Notably, among 321 “IM Genes,” only 205 genes (63.9%) were upregulated, and 116 genes (36.1%) were downregulated in the DOX(+) organoids (Figure 5 upper right panel). The statistical analysis indicated that the proportion of CDX2-upregulated genes among “IM Genes” was significantly lower than that among “Intestinal Genes” (Figure 5 lower panels).

We then showed the individual experimental data for each of the 71 “Intestinal Genes” as a heatmap (Figure S10). Eight genes (BEST4, GUCA2A, CA7, RNF186, TMIGD1, MYO7B, OTOP3, and CDX2) were more than 5-fold upregulated on average in DOX(+) samples compared to DOX(−) ones. Notably, all eight genes were highly expressed in Paneth cells (Karlsson et al., 2021), suggesting that some mechanism to direct the cell fate toward Paneth cells might be activated after CDX2 induction.

### Expression of the master transcription factors CDX1, CDX2, SOX2, and GATA4

**Figure 6:**
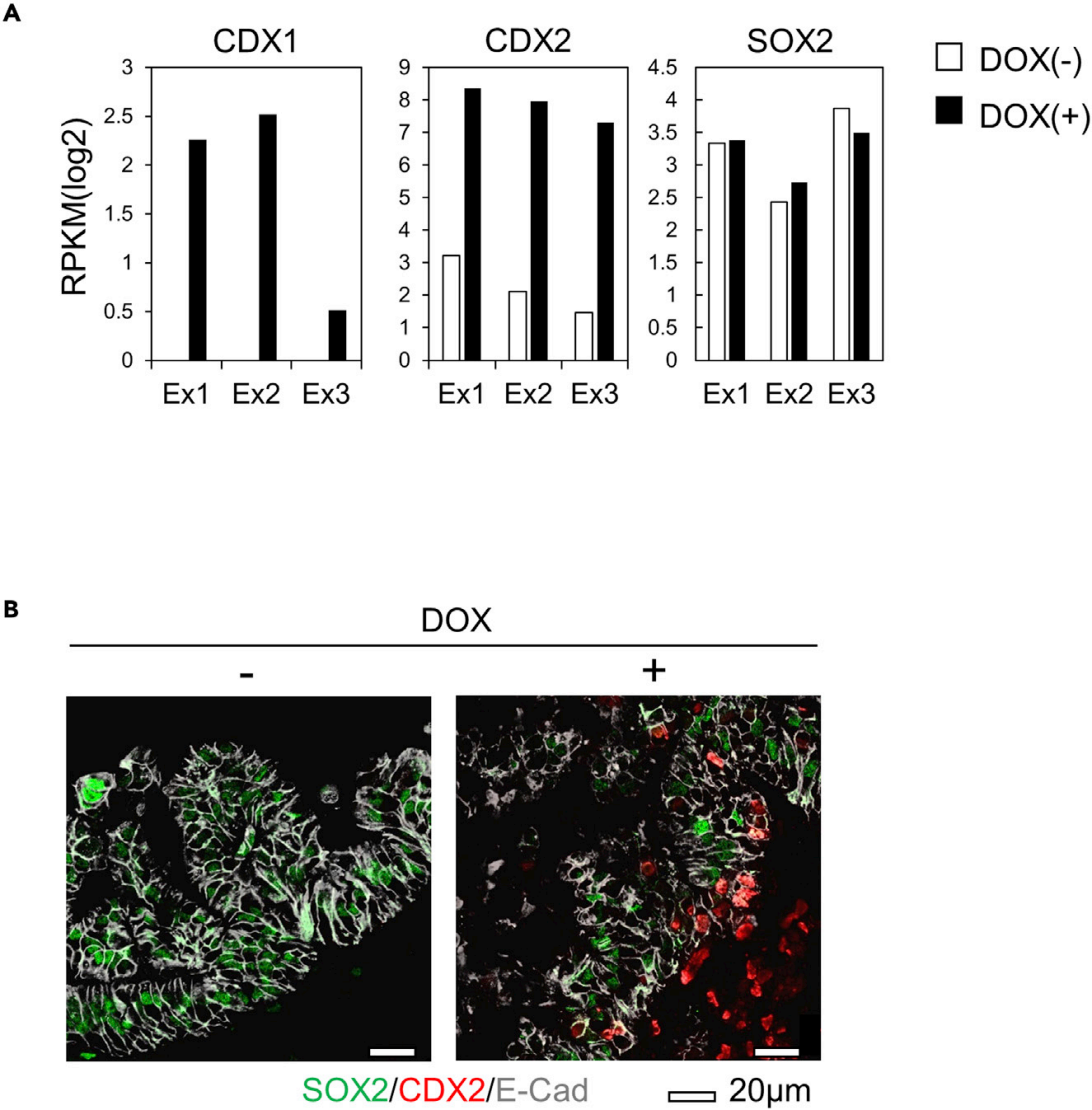
The expression of the master transcription factors CDX1, CDX2, and SOX2 (A) RPKM values (log2) of CDX1, CDX2, and SOX2 in RNA-seq data. (B) Immunofluorescence analyses of E-Cadherin, SOX2, and CDX2 in gastric organoids from CDX2-iPSC at Day 40 without (−) (left panel) or with (+) (right panel) DOX treatment for 5 days, using confocal microscopy. Scale bars, 20 μm.

The intestinal master transcription factor CDX1 was upregulated in DOX(+) organoids compared to DOX(−) ones on RNA-seq analysis (Figure 6A, left panel). Total CDX2 was obviously upregulated in DOX(+) organoids than in DOX(−) ones on RNA-seq analysis (Figure 6A, middle panel).

In RNA-seq analysis of whole organoid samples, no obvious difference in the expression of SOX2, a gastric epithelial transcription factor found between the DOX(+) and DOX(−) organoids (fold change: 0.77-1.24) (Figure 6A, right panel); however immunofluorescence demonstrated the disappearance of SOX2 expression on the CDX2-overexpressing epithelium, indicated by the expression of E-cadherin in DOX(+) organoids (Figures 6B and S6F). This finding suggested that the expression of CDX2 suppresses the expression of SOX2 on the gastric epithelium.

GATA4 is a transcription factor expressed in both stomach and intestine tissues (Uhlen et al., 2015) and was reported to regulate proliferation in the early developing intestine (Kohlnhofer et al., 2016) and to control intestinal crypt cell replication in conjunction with CDX2 (San Roman et al., 2015). Consistent with these previous findings, GATA4 expression was widely observed in stomach tissues, the DOX (−) gastric organoids and the DOX (+) organoids (Figure S11).

## Discussion

In the present study, we established an *in vitro* human incomplete intestinal metaplasia model by overexpressing CDX2 in human gastric organoids derived from iPSC. This model represented (i) the upregulation and downregulation of well-known intestinal and gastric marker genes at the mRNA level, respectively, (ii) the appearance of cells positive for the intestinal markers CK20 and MUC2, (iii) the disappearance of cells expressing MUC5AC, a gastric foveolar cell marker, (iv) the appearance of Crypt-like structure and MUC2-positive cell clusters in the structure, and (v) the emergence of a Paneth cell gene expression signature. Several animal models of gastric intestinal metaplasia (Honda et al., 1998; Mutoh et al., 2002; Silberg et al., 2002; Zheng et al., 2004) and the induction of the intestinal phenotype in human gastric cancer cell lines and an immortalized gastric epithelial cell line (Fujii et al., 2012) have been reported. However, an intestinal metaplasia model using human gastric epithelial tissues has yet to be established. Species differences in the pathogenesis of various diseases remain a subject of debate (Nature Medicine Editorial, 2013; Seok et al., 2013; Takao and Miyakawa, 2015). In addition, in *H. pylori*-related gastric carcinogenesis, not only the direct pathogenicity of *H. pylori* against gastric epithelial cells but also its indirect pathogenicity via immune-mediated inflammation is important (Chiba et al., 2006), and our *in vitro* system might be able to mimic various inflammatory microenvironments around gastric epithelial tissue by adding cytokines or immune cells to the culture media. Although the eradication of *H. pylori* is widely performed today and reportedly contributes to a reduction in gastritis and improvement in gastric atrophy, IM is irreversible even after *H. pylori* eradication (Chen et al., 2020), and the recurrence rate of *H. pylori* infection has been increasing in recent years (Zhao et al., 2021). In addition, intestinal metaplasia was recently reported to be induced by not only *H. pylori* infection but also bile acids (Li et al., 2019; Xu et al., 2020; Yu et al., 2019; Yuan et al., 2019). Therefore, it is still important to study the pathogenesis of IM and ways to restore the gastric mucosa. The model established in this study can help clarify the molecular mechanisms underlying gastric carcinogenesis, leading to the development of novel therapies and/or prevention strategies for gastric cancer.

Our present model of human gastric intestinal metaplasia exhibited the downregulation of SOX2, a “master transcription factor” that determines the cell identity for the upper gastrointestinal tract (Que et al., 2007). Metaplasia is generally understood to be the consequence of a change in the expression of the master transcription factor for one tissue to that of another (Slack, 2009); in the case of gastric intestinal metaplasia, this involves changing from SOX2 to CDX2. Indeed, several reports have shown that the SOX2 expression decreased under conditions of intestinal metaplasia (Niu et al., 2017; Tsukamoto et al., 2005, 2006), although another report argued that the Sox2 expression increased in the intestinal metaplastic mucosa of Cdx2-transgenic mouse stomach (Mutoh et al., 2011). In the gastric epithelium, *H. pylori* infection was reported to cause upregulation of the CDX2 expression concomitantly with SOX2 downregulation (Chen et al., 2020), but the data in our present study suggest that the enhanced expression of CDX2 in the gastric epithelium may suppress the expression of SOX2. Consistent with our findings, an inverse correlation between SOX2 and CDX2 was recently reported in gastric cancer and colorectal cancer (Helal et al., 2020; Lopes et al., 2020), suggesting the mutual suppression of the expression of SOX2 and CDX2. Of note, the endogenous CDX2 gene was not upregulated in our model or in the Cdx2-transgenic mouse model (Mutoh et al., 2009), indicating that neither model has the ability to recapitulate the changes from the gastric transcriptional network to the autonomous intestinal transcriptional network. Moreover, this RNA-seq analysis showed the significant upregulation of CDX1. A previous report argued that the expression of CDX1 plays an important role in intestinal metaplasia in the gastric epithelium (Mutoh et al., 2004). In the case of the generation of iPSC from gastric epithelial cells, the expression of transgenes are only required for induction but not for the maintenance of a pluripotent state (Aoi et al., 2008). This suggests that gastric epithelial cells can be changed into other types of cells, including intestinal cells, in which a master transcriptional network is established and thereafter maintained without the persistent expression of transgenes.

The genome-wide transcription analysis in the present study showed that >85% of “Intestinal Genes” were upregulated in the gastric organoids by the overexpression of CDX2.

Notably, the proportion of CDX2-upregulated genes among “IM Genes” was significantly less than that among “Intestinal Genes.” These results suggested two hypotheses: (1) IM, which is clinically observed and which is an important issue in the context of gastric carcinogenesis, is a different phenomenon from mere “fate conversion of stomach into intestine,” (2) the forced expression of CDX2 alone is not enough to induce *bona-fide* intestinal metaplasia in the gastric epithelium, and some other critical trigger is required. Based upon the system that we established in this study and hypothesis, we would be able to unravel the suppressive mechanisms of cell fate conversion and the reasons for the failure of the mechanisms. Furthermore, the overexpression of some additional factors or different culture conditions may enable us to establish a model that fully recapitulates the cell fate change from a gastric identity to an intestinal one.

### Limitations of the study

In contrast to previously reported mouse intestinal metaplasia models (Silberg et al., 2002), our present model did not show the obvious upregulation of several conventional markers for intestinal metaplasia, such as MUC2 and villin (Park et al., 2010; Reis et al., 1999), in RNA-seq analysis, and only a few MUC2-positive cells were found in immunofluorescence. The phenotype of the stomach in previously reported Cdx2-transgenic mouse models was analyzed at 1 to 15 weeks of age (Silberg et al., 2002), whereas we analyzed the phenotype 5 to 12 days after CDX2 overexpression in the present study. This difference in the period of assessing CDX2 overexpression may have resulted in the observed differences in the marker gene expression pattern. We applied gastric organoid culture medium (McCracken et al., 2014), but not intestinal medium, to both DOX(−) and DOX(+) organoids in the present study. Our present results are consistent with those of a previous report on mouse tissue-derived gastric organoids artificially expressing CDX2, which were cultured for more than 35 days under gastric organoid culture conditions (Simmini et al., 2014). Crafting intestinal organoid culture conditions containing Noggin, R-Spondin, and Wnt3a (Fordham et al., 2013; Sato et al., 2009) might enable the maintenance of CDX2-overexpressing organoids for an extended period of time, thereby allowing us to obtain a complete intestinal phenotype.

## STAR★Methods

### Key resources table

**Table:** 

REAGENT or RESOURCE	SOURCE	IDENTIFIER
**Antibodies**		

mouse monoclonal anti-OCT3/4	BD Biosciences	Cat# 611202; RRID: AB_398736
rabbit polyclonal anti-SOX2	Abcam	Cat# ab97959; RRID: AB_2341193
rabbit monoclonal anti-SOX2	Abcam	Cat# ab93689; RRID: AB_10562630
mouse monoclonal anti-CDX2	Thermo Fisher Scientific	Cat# 39-7800; RRID: AB_2533435
mouse monoclonal anti-MUC5AC	Leica Biosystems	Cat# NCL-MUC-5AC; RRID: AB_442113
mouse monoclonal anti-MUC2	Leica Biosystems	Cat# NCL-MUC-2; RRID: AB_442112
rat monoclonal anti-E-cadherin	Takara	Cat# M108; RRID: AB_2895157
goat polyclonal anti-NANOG	R&D Systems	Cat# AF1997; RRID: AB_355097
goat polyclonal anti-SOX17	R&D Systems	Cat# AF1924; RRID: AB_355060
mouse monoclonal anti-α-SMA	Dako	Cat# M0851; RRID: AB_2223500
mouse monoclonal anti-β-III tubulin	Millipore	Cat# MAB1637; RRID: AB_2210524
mouse monoclonal anti-GATA4	Santa Cruz Biotechnology	Cat# sc-25310; RRID: AB_627667
mouse monoclonal anti-E-cadherin (E-cad)	Thermo Fisher Scientific	Cat# 33-4000; RRID: AB_2533118
goat polyclonal anti-E-cad	R&D systems	Cat# AF648; RRID: AB_355504
mouse monoclonal anti- cytokeratin 20 (CK20)	Dako	Cat# IR777; RRID: AB_2133718
mouse monoclonal anti- cytokeratin 7 (CK7)	Dako	Cat# M7018; RRID: AB_2134589
mouse monoclonal anti-CDX2	Biocare Medical	Cat# CM226-C; RRID: AB_2335616
rabbit monoclonal anti-SOX2	Thermo Fisher Scientific	Cat# MA5-16399; RRID: AB_2537918
goat polyclonal anti-PDX1	Abcam	Cat# ab47383 RRID: AB_2162359
rabbit polyclonal anti-Somatostatin	Dako	Cat# A0566; RRID: AB_2688022
mouse monoclonal anti-H, K-ATase	MBL International	Cat# D031-3; RRID: AB_590576
rabbit monoclonal anti-Synaptophysin	Roche	Cat# 760-4595; RRID: AB_2857955
mouse monoclonal anti-Chromogranin A	DAKO	Cat# M0869; RRID: AB_2081135
mouse monoclonal anti-β-actin	Sigma Aldrich	Cat# A5441; RRID: AB_476744

**Chemicals, peptides, and recombinant proteins**		

iMatrix-511	Nippi	Cat# 892-021
Stem Fit	Ajinomoto	
penicillin and streptomycin	Life Technologies	Cat# 15140-122
TrypLE Select	Life Technologies	Cat# A12177-01
Rock inhibitor(Y-27632)	WAKO	Cat# 253-00513
Fugene HD	Promega	Cat# E2311
G418	Nacalai Tesque	Cat# 16512-36
Activin A	Peprotech	Cat# 120-14-250UG
BMP4	Peprotech	Cat# 314-BP
B27	Life Technologies	Cat# 17504-001
L-glutamine	Life Technologies	Cat# 25030-081
FBS	BIOWEST	Cat# F7524
CHIR99021	TOCRIS	Cat# 4423
FGF4	Cell Guidance Systems	Cat# GFH31
NOGGIN	R&D Systems	Cat# 6057-NG
HEPES	Invitrogen	Cat# 15630-080
EGF	R&D Systems	Cat# 236-EG
Retinoic acid	WAKO	
N2	Life Technologies	Cat# 17502-001

**Critical commercial assays**		

Prime Script II 1st strand cDNA Synthesis Kit	Takara	Cat# 6210B
Takara Ex Taq PCR kit	Takara	Cat# RR001A
TURBO DNA-free kit	Thermo Fisher Scientific	Cat# AM1907
TB Green Premix Ex Taq II	Takara	Cat# RR420A
LR clonase II	Life Technologies	Cat# 11791-020
XT ultraView Universal DAB Detection Kit	Ventana Medical Systems, Inc.	Cat# 760-500

**Deposited data**		

RNA-seq	This paper	GEO: GSE173624
Listing genes highly expressed in the intestine	Fagerberg et al. (2014)	Array Express Archive (www.ebi.ac.uk/arrayexpress/) under the accession number E-MTAB-1733
the Human Protein Atlas	Karlsson et al. (2021)	https://www.proteinatlas.org

**Experimental models: Cell lines**		

iPSC line (201B7)	Riken BRC (Tsukuba, Japan)	HPS0001, RRID:CVCL_A324
iPSC line (FF-PB-3AB4)	Suzuki et al. (2019)	
iPSC line (CDX2-iPSC)	This paper	
Colo320 DM	The Japanese Collection of Research Bioresources (JCRB) Cell bank (Osaka, Japan)	JCRB0225, RRID:CVCL_0219

**Oligonucleotides**		

human CDX2 cloning primer, forward primer 5′-CACCATGTACGTGAGCTACCTCCTGGACAAGGAC-3′	This paper	
human CDX2 cloning primer, reverse primer 5′-TCAC TGGGTGACGGTGGGGTTTAGCACCCCCCCAGTTG-3′	This paper	
Primers for hGAPDH, see Table S1	Okita et al. (2011)	
Primers for hOCT3/4, see Table S1	Takahashi et al., 2007	
Primers for hSOX2, see Table S1	Takahashi et al., 2007	
Primers for hNANOG, see Table S1	Takahashi et al., 2007	
Primers for hCDX2, see Table S1	This paper	
Primers for hPDX1, see Table S1	This paper	
Primers for hMUC5AC, see Table S1	This paper	
Primers for hCK7, see Table S1	This paper	
Primers for hCK20, see Table S1	This paper	
Primers for hSOX17, see Table S1	This paper	
Primers for hFOXA2, see Table S1	This paper	

**Recombinant DNA**		

pENTR/D-TOPO	Life Technologies	
PB-TAC-ERN	Kim et al. (2016)	provided by Dr. Knut Woltjen at Kyoto University
pCAG-PBase	Kim et al. (2016)	provided by Dr. Knut Woltjen at Kyoto University

**Software and algorithms**		

Strand NGS software program	Agilent	
JSTAT 6.5		http://toukeijstat.web.fc2.com/

### Resource availability

#### Lead contact

Further information and requests for resources and reagents should be directed to and will be fulfilled by the lead contact, Takashi Aoi (takaaoi@med.kobe-u.ac.jp).

#### Materials availability

This study did not generate new unique reagents.

### Experimental model and subject details

#### iPSC culture

The validated iPSC line 201B7 was purchased from Riken Cell bank (Tsukuba, Japan) and transferred from on-feeder to feeder-free conditions in our laboratory. We cultured the iPSC lines according to a previously described method (Nakagawa et al., 2014). In brief, the culture plates were precoated with iMatrix-511 (0.5 μg/cm^2^), and the iPSC were maintained in Stem Fit medium (Ajinomoto) with penicillin (100 units/mL) and streptomycin (100 μg/mL; Life Technologies, MA, USA) at 37°C with 5% CO_2_. The medium was changed every other day and passaged every 7-10 days using 0.5× TrypLE Select (1× TrypLE Select diluted 1:1 with 0.5 mM EDTA/PBS [-]; Life Technologies) and Rho-associated kinase (Rock) inhibitor (Y-27632; WAKO, Osaka, Japan).

### Method details

#### Vector construction and generation of CDX2-iPSC

The cDNA encoding human CDX2 open reading frame (ORF) was amplified by polymerase chain reaction (PCR) using the forward primer 5′-CACCATGTACGTGAGCTACCTCCTGGACAAGGAC-3′ and reverse primer 5′-TCACTGGGTGACGGTGGGGTTTAGCACCCCCCCAGTTG-3′, and the resulting PCR product was cloned into pENTR/D-TOPO (Life Technologies) to generate pENTR-CDX2 clone according to the manufacturer’s protocol. LR clonase II (Life Technologies) recombination was then performed using a pENTR-CDX2 clone and the destination vector PB-TAC-ERN (Kim et al., 2016) to generate PB-TAC-CDX2-ERN.

To generate CDX2-iPSC, the dissociated single cells of FF-PB-3AB4 were seeded onto an iMatrix-511coated 6-well plate at a density of 5 × 10^5^ cells/well. The next day, the cells were transfected with 1.5 μg of pCAG-PBase (Kim et al., 2016) and 1.5 μg of PB-TAC-CDX2-ERN using Fugene HD (Promega, WI, USA). Forty-eight hours after transfection, 100 μg/mL G418 (Nacalai Tesque, Kyoto, Japan) was added to select transduced cells for 4 days. After 8 hiPSC colonies were isolated and expanded, the subclone CDX2-iPSC that strongly expressed mCherry by DOX addition (1 μM for 3 days) was selected and used in this paper. G418 (100 μg/mL) was continuously added during the maintenance culture of CDX2-iPSC before differentiation into gastric organoids.

#### Gastric organoid differentiation

Differentiation into gastric organoids was performed according to a previously reported method (McCracken et al., 2014). In brief, 2.5 × 10^5^ to 4.0 × 10^5^ iPSC were plated as single cells in a 12-well dish (Nunc Cell-Culture Treated Multidishes; Thermo Scientific, MA, USA) in StemFit medium with 10 μM of Rock inhibitor Y-27632 (WAKO). The next day, the medium was changed to RPMI-1640 medium (Nacalai Tesque) supplemented with 100 ng/mL of Activin A (Peprotech, NJ, USA), 50 ng/mL of BMP4 (Peprotech), B27 (Life Technologies), L-glutamine (Life Technologies), penicillin and streptomycin. Activin A and B27 and L-glutamine were added for three days, and BMP4 was added on the first day. FBS (BIOWEST, Nuaillé, France) contained increasing concentrations of 0%, 0.2 and 2.0% to induce differentiation into DE by Day 4.

To generate foregut spheroids, RPMI1640 medium supplemented with 2% FBS, 2 μM of CHIR99021 (TOCRIS, Bristol, UK), 500 ng/mL of FGF4 (Cell Gaidance, MO, USA), 200 ng/mL of NOGGIN (R&D Systems, MN, USA), penicillin and streptomycin was added for 3 days until Day 7. Retinoic acid (2 μM; WAKO) was added on Day 6.

For three-dimensional culture of gastric organoids, we used Matrigel (354234; CORNING, NY, USA), advanced DMEM/F12 medium (Life Technologies), N2 (Life Technologies), B27, L-glutamine, 10 mM of HEPES (Invitrogen, MA, USA), penicillin/streptomycin and 100 ng/mL of EGF (R&D Systems) at Day 7. For the first 3 days, 2 μM retinoic acid and 200 ng/mL NOGGIN were added to the media. The media were replaced every three to four days, as necessary.

#### A semi-quantitative or real-time quantitative reverse-transcriptase (RT)-PCR analysis

Total RNA was isolated using Trizol (Life Technologies) and treated with the TURBO DNA-free kit (Thermo Fisher Scientific). For the extraction of RNA from CDX2-iPSC derived DOX(+)/(−) gastric organoids, Invitrogen Phasemaker Tubes (Thermo Fischer Scientific) were used with Trizol, and DNase treatment was not performed. The Prime Script II 1st strand cDNA Synthesis Kit (Takara, Shiga, Japan) was used to synthesize cDNA from 200-500 ng of total RNA.

For semi-quantitative RT-PCR analysis (Figures 1C, 2B, 3D, and S1B), the resulting cDNA was subjected to PCR with a TaKaRa Ex Taq® PCR kit (Takara). For the quantitative PCR analysis (Figures 4C and S2B), we used a Light Cycler®480 Real time PCR system (Roche) with TB Green® Premix Ex Taq™ II (Takara). The PCR primers used are listed in Table S1.

#### Western blotting

After 1 μM DOX treatment of CDX2-iPSC for 2 days, the cells were washed once with PBS, lysed with the M-PER Mammalian Protein Extraction Reagent (Thermo Fisher Scientific) and subjected to SDS-polyacrylamide gel electrophoresis (SDS-PAGE). After the electrophoretic transfer of the proteins to the PVDF membranes, immunoblotting with mouse anti-CDX2 (Invitrogen) and mouse anti-β-actin (Sigma Aldrich, MO, USA) followed by horseradish peroxidase (HRP)-conjugated secondary antibodies at a 1:3000 dilution in Can Get Signal immunoreaction enhancer solution (TOYOBO, Osaka, Japan) was performed. The Amersham Imager 600 imagers (Cytiva, Tokyo, Japan) was used to detect signals.

#### Immunocytochemistry

Cells were fixed with PBS containing 4% paraformaldehyde for 10 min at room temperature. After washing with PBS, the cells were treated with 0.3% Triton X-100 in PBS for 45 min at room temperature and blocked with 2% skim-milk in PBS for 1 h. The cells were incubated with primary antibodies at 4°C overnight and then stained with secondary antibodies. The primary antibodies were mouse anti-OCT3/4 (611202, dilution 1:200; BD transduction Laboratories, New Jersey, USA), rabbit anti-SOX2 (ab97959, dilution 1:100; abcam, Cambridge, UK), rabbit anti-SOX2 (ab93689, dilution 1:100; abcam), mouse anti-CDX2 (39-7800, dilution 1:100; Invitrogen), mouse anti-MUC5AC (CLH2, dilution 1:100; Leica, Nussloch, Germany), anti-MUC2 mouse monoclonal antibody (Ccp58, dilution 1:200; Leica), rat anti-E-cadherin (M108, dilution 1:500; Takara), goat anti-NANOG (AF1997, dilution 1:200; R&D Systems), goat anti-SOX17 (AF1924, dilution 1:200, R&D Systems), mouse anti-α-SMA (M0851, dilution 1:200; Dako, Glostrup, Denmark), mouse anti-β-III tubulin (MAB1637, dilution 1:200; Millipore, MA, USA) and anti-GATA4 mouse monoclonal antibody (sc-25310, dilution 1:200; Santa Cruz, TX, USA). All of the secondary antibodies (Alexa Fluor 594-conjugated anti-mouse or anti-goat IgG, Alexa Fluor 488-conjugated anti-mouse, anti-goat or anti-rat; or anti-rabbit IgG, Cy5 647-conjugated anti-mouse IgG) were obtained from Life Technologies. Hoechst 33342 (WAKO) was used for nuclear staining. In Figures 2D, 3B, 3E, 4E, 4F, 6B, S7B and S11, representative data of two or three independent experiments are shown.

#### *In vitro* spontaneous differentiation via embryoid body formation

For embryoid body (EB) formation, undifferentiated iPSC were dissociated into single cells, resuspended in Primate ES medium (Reprocell) containing 20 μM Rock inhibitor Y-27632 (WAKO) and seeded on low-cell-adhesion 96-well spindle-bottom plates (PrimeSurface, Sumitomo Bakelite, MS-9096M; Tokyo, Japan) at 1 × 10^4^ cells per well. After 7 days of culture, the EBs were transferred to gelatin-coated 24-well plates and cultured in the same medium for another 7 days. The differentiated cells were immune-stained with the indicated antibodies.

#### Karyotype analyses

The G-band karyotype analysis for FF-PB-3AB4 was performed at Chromocenter, Inc. (Yonago, Japan).

#### Frozen section samples

The cultured organoids were fixed with 4% paraformaldehyde and then embedded in Tissue-Tek O.C.T Compound (Sakura Finetek Japan, Tokyo, Japan) and frozen at −80°C. The frozen samples were sectioned at 5-8 μm on a cryostat.

#### Histological and immunohistochemical analyses of the organoids

The organoids were embedded in paraffin blocks and sectioned at 4-μm thickness. The sections were deparaffinized and stained with Hematoxylin and Eosin (HE). Immunohistochemistry was performed using the Benchmark XT (Roche, Basel, Switzerland) autostainer with an XT ultraView Universal DAB Detection Kit (Ventana Medical Systems, Inc., AZ, USA).

For immunofluorescence, primary antibodies were incubated overnight at 4°C. Slides were washed in PBS and incubated with secondary antibody for 1 h at room temperature. For paraffin embedded sections, antigen retrieval was performed with 1 mM EDTA (pH8.0) for 3 min in pressure cooker before primary antibody incubation.

The primary antibodies used in this study are listed below. anti-E-cadherin (E-cad) mouse monoclonal antibody (Clone: 4A2C7, dilution 1:100, Zymed, CA, USA), anti-E-cad polyclonal goat antibody (Catalog Number: AF648, dilution1:200, R&D systems, Figure 3B), anti-E-cad rat monoclonal antibody (Clone: ECCD-2, dilution 1:1000, Takara, Figure 4F), anti- cytokeratin 20 (CK20) mouse monoclonal antibody (Clone: Ks20.8, dilution 1:50; Dako), anti- cytokeratin 7 (CK7) mouse monoclonal antibody (Clone: OV-TL 12/30, dilution 1:50; Dako), anti-CDX2 mouse monoclonal antibody (CM226, dilution 1:50; Biocare Medical, CA, USA), anti-CDX2 mouse monoclonal antibody (ZC007, dilution 1:100, invitrogen), anti-SOX2 rabbit monoclonal antibody (SP76, dilution 1:100; Invitrogen), anti-PDX1 goat polyclonal antibody (dilution 1:100; abcam), anti-MUC5AC mouse monoclonal antibody (CLH2, dilution 1:100; Leica), anti-MUC2 mouse monoclonal antibody (Ccp58, dilution 1:200; Leica), anti-Somatostatin rabbit monoclonal antibody (A0566, dilution 1:800; DAKO), anti-H, K-ATase mouse monoclonal antibody (Clone: 1H9, dilution 1:2000; MBL, Osaka, Japan), anti-Synaptophysin rabbit monoclonal antibody (Clone: MRQ-40, dilution 1:100; Roche) and anti-Chromogranin A mouse monoclonal antibody (Clone: DAK-A3, dilution 1:200; DAKO). Secondary antibodies and hoechst for immunofluorescence were same as used in immunocytochemistry. In Figure S5A and S5B, representative data of three or more independent experiments are shown.

#### RNA sequencing

Total RNA was isolated using Trizol (Life Technologies) and treated with the TURBO DNA-free kit (Thermo Fisher Scientific), as described above. The RNA was sent to Macrogen (Seoul, South Korea, https://www.macrogen.com) for library preparation and paired-end RNA sequencing on the Illumina Novaseq6000 platform. Raw sequence files (fastq) were aligned to the human transcriptome (hg38) reference sequences using the Strand NGS software program (Strand Life Science, Karnataka, India) with default parameters. The aligned reads were normalized using Reads per kilobase of exon per million mapped reads (RPKM) again with the Strand NGS software program. For analysis, only the genes whose RPKM values are more than 0.1 (log2) in at least one sample of six samples were used to filter out noise from the expression data. The total number of genes used in the analysis was 15511. RNA-seq data have been deposited in the Gene Expression Omnibus (GEO) under accession number GSE173624.

#### Listing genes highly expressed in the intestine (colon, small intestine, or duodenum) compared to the stomach

According to the protocol of a previous report (Fagerberg et al., 2014), we obtained the RNA-seq data of 14 samples from stomach and 3 types of tissue in the intestine, including 3 from the stomach, 5 from the colon, 4 from the small intestine and 2 from the duodenum through the Array Express Archive (www.ebi.ac.uk/arrayexpress/) under the accession number E-MTAB-1733. The average fragments per kilobase of exon per million mapped fragments (FPKM) values of all individual samples from each tissues were used to estimate the gene expression. We defined genes highly expressed in the intestine compared to the stomach as follows: (1) >10 FPKM in at least 1 tissue of the intestine; (2) >50-fold higher FPKM in at least 1 tissue of the intestine compared to the stomach. A total of 172 ensemble genes IDs met these criteria.

### Quantification and statistical analysis

#### Statistical analyses

Statistical analyses were conducted using an unpaired two-tailed Student’s *t* test and Pearson’s chi-square test. p values <0.05 were considered significant. All data were analyzed using the Jstat software program (JSTAT 6.5, http://toukeijstat.web.fc2.com/).

## Data Availability

• RNA-seq data have been deposited at GEO and are publicly available as of the date of publication. • This paper does not report original code. • Any additional information required to reanalyze the data reported in this paper is available from the lead contact upon reasonable request.
