# Supplementary material for: CDX2-induced intestinal metaplasia in human gastric organoids derived from induced pluripotent stem cells
Source: iScience. 2022 Apr 28;25(5):104314. doi: 10.1016/j.isci.2022.104314 (PMC9118752; doi:10.1016/j.isci.2022.104314)
Supplement: Document S1. Figures S1–S11 and Table S1 [file mmc1.pdf]

iScience, Volume *25*

Supplemental information

CDX2-induced intestinal metaplasia in human gastric organoids derived from induced pluripotent stem cells

Takahiro Koide, Michiyo Koyanagi-Aoi, Keiichiro Uehara, Yoshihiro Kakeji, and Takashi Aoi

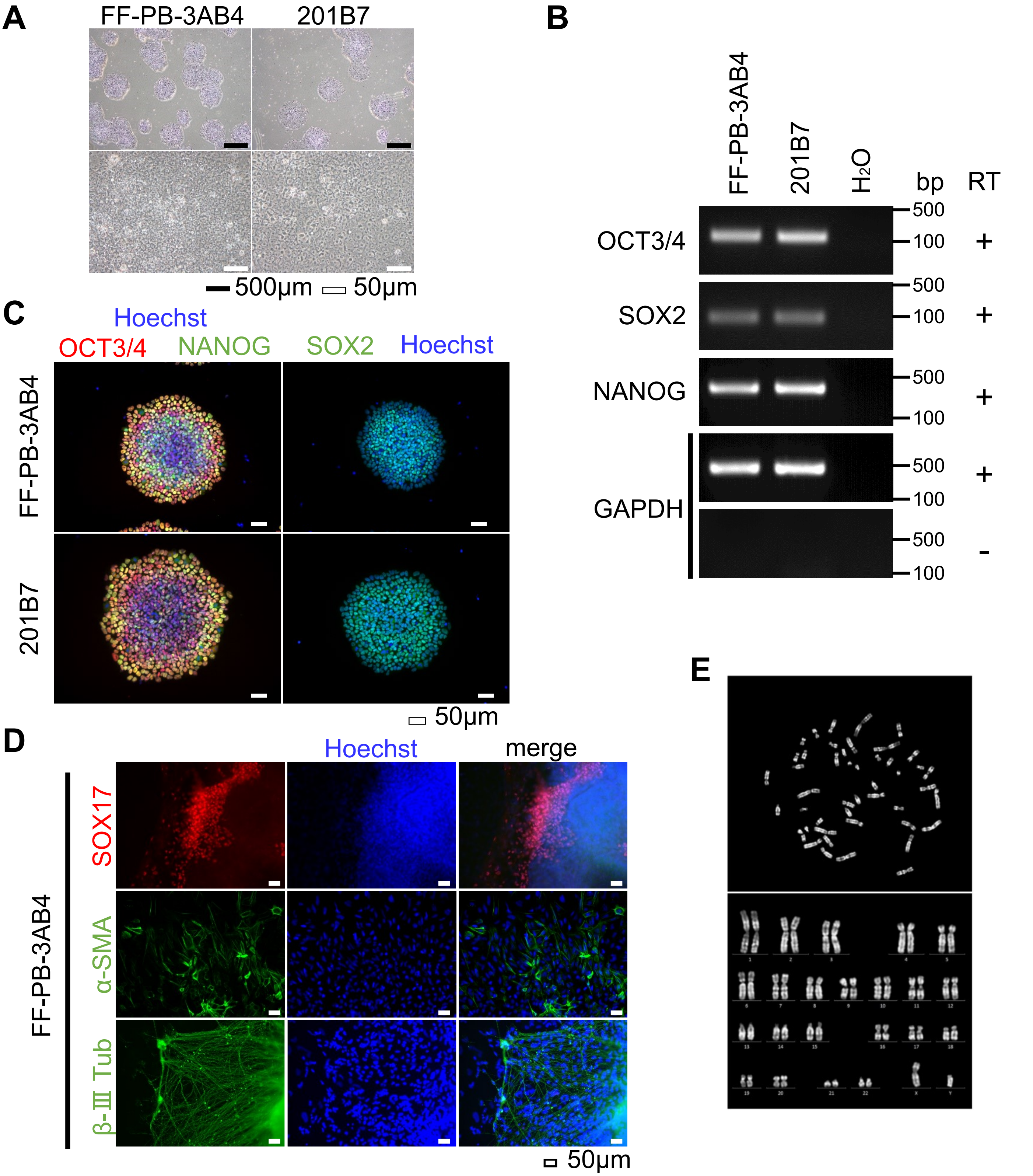

Figure S1. The validation of the hiPSC line FF-PB-3AB4, Related to Figure 1. A. Cell morphologies of FF-PB-3AB4 (left panels) and the reference iPSC line 201B7 (right panels) Scale bars: black bar = 500 μm, white bar = 50 μm. **B**. An RT-PCR analysis showed that FF-PB-3AB4 expressed the pluripotent markers OCT3/4, SOX2 and NANOG. GAPDH was used as an endogenous control. RT: reverse transcriptase. **C**. Immunostaining showed that FF-PB-3AB4 as well as 201B7 expressed the pluripotent markers OCT3/4 and NANOG (left panels) and SOX2 (right panels). Hoechst 33342 dye was used to stain nuclei (blue). Scale bars, 50 μm. **D**. FF-PB-3AB4 generated cell types of all three embryonic germ layers: SOX17 for endoderm, α-smooth muscle actin (α-SMA) for mesoderm and beta-III tubulin (β-III Tub) for ectoderm *in vitro* via the formation of an embryoid body. Scale bars, 50 μm. **E**. The G-banded karyotype indicated normal diploid male chromosomal content (46XY).

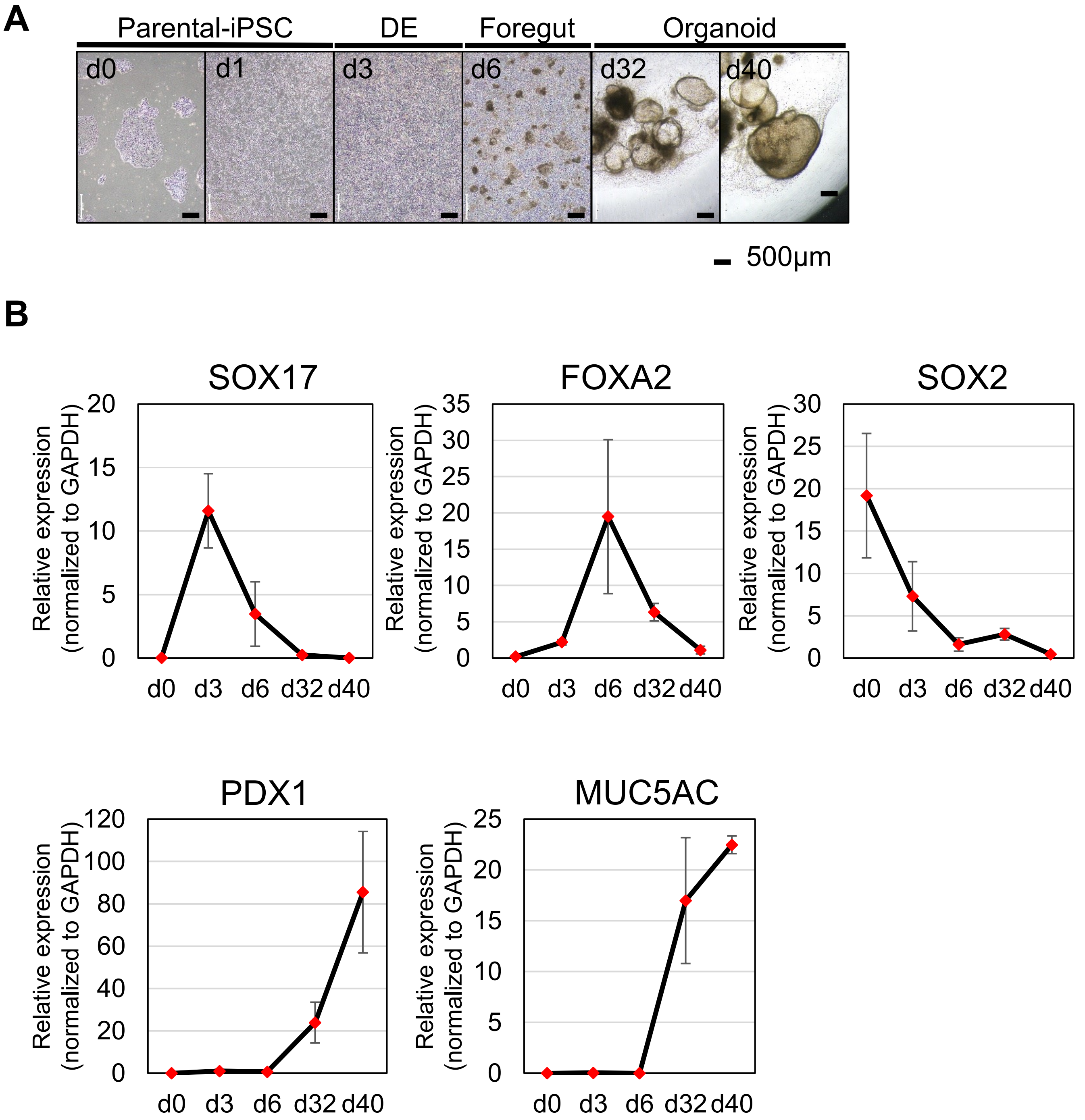

Figure S2. Gastric organoid differentiation, Related to Figure 3. A. Cell morphologies of iPSC (d0, d1), definitive endoderm (DE, d3), foregut (d6) and gastric organoids (d32, d40). Scale bars, 500 μm. **B**. qRT-PCR analysis of differentiated markers (SOX17, FOXA2, SOX2, PDX1 and MUC5AC) in iPSC (d0), DE (d3), foregut (d6) and organoids (d32, d40). The mRNA expression was normalized to GAPDH. Data are represented as mean +/- SEM of three independent induction experiments.

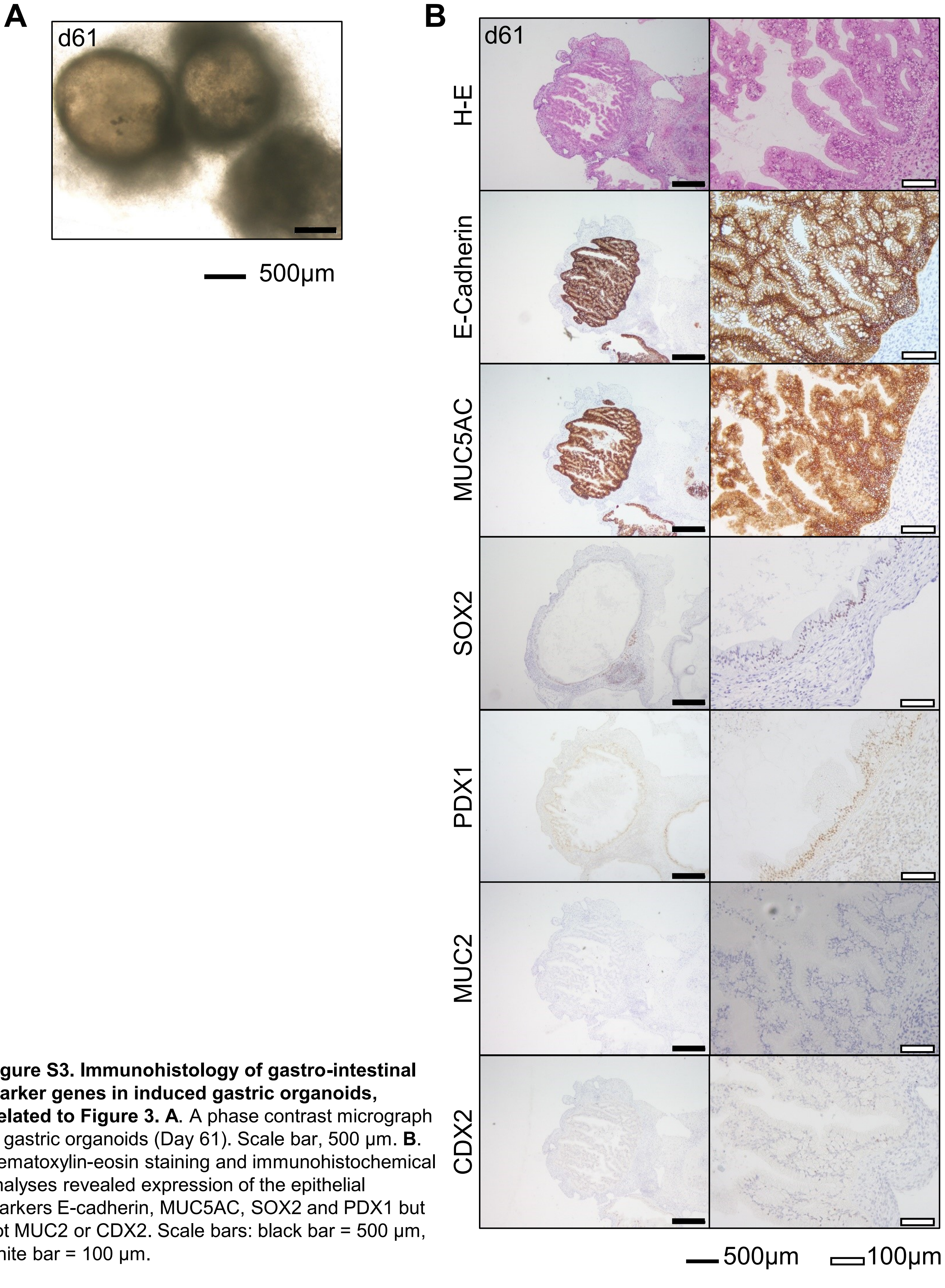

Figure S3. Immunohistology of gastro-intestinal marker genes in induced gastric organoids, Related to Figure 3. A. A phase contrast micrograph of gastric organoids (Day 61). Scale bar, 500 µm. **B**. Hematoxylin-eosin staining and immunohistochemical analyses revealed expression of the epithelial markers E-cadherin, MUC5AC, SOX2 and PDX1 but not MUC2 or CDX2. Scale bars: black bar = 500 µm, white bar = 100 µm.

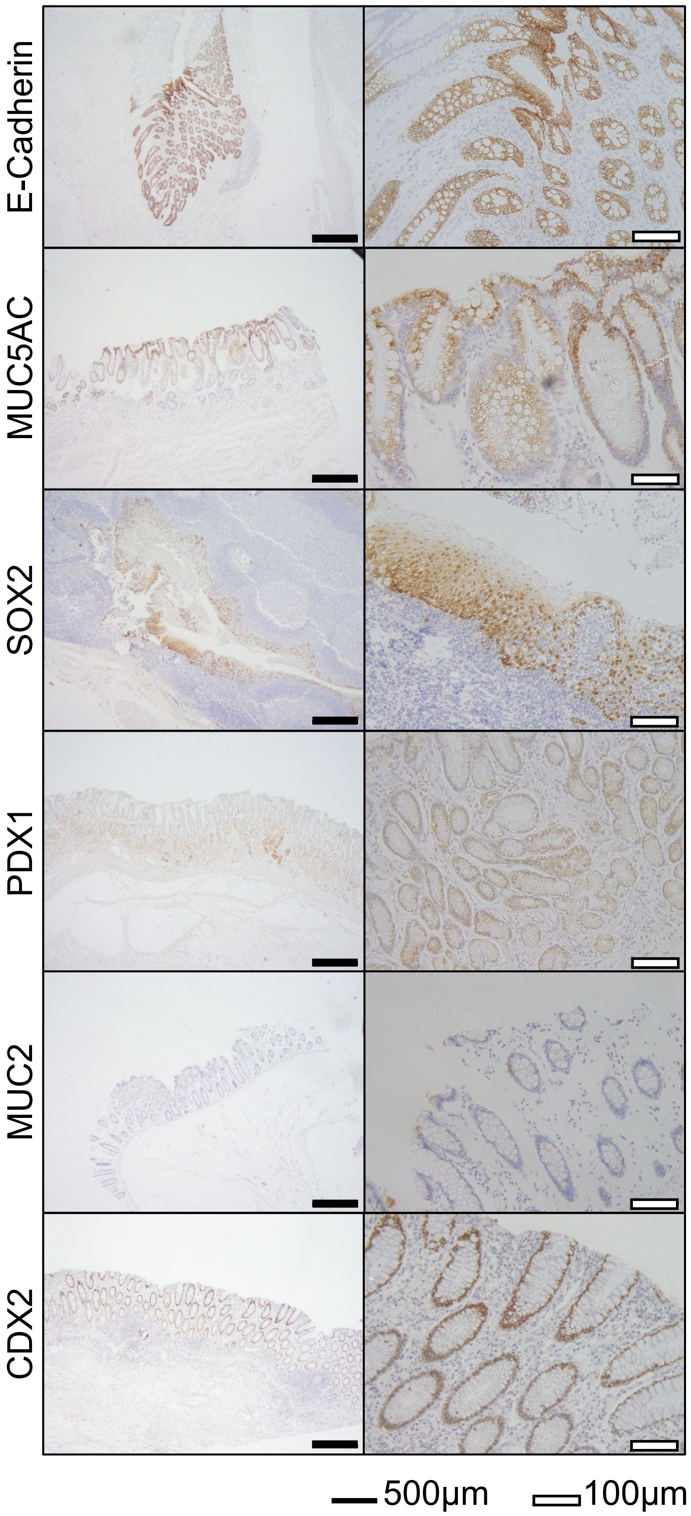

Figure S4. Positive controls of immunohistologies for gastro-intestinal marker genes in induced hGOs, Related to Figure S3. E-Cadherin, MUC5AC and PDX1 were positive in human stomach tissues, MUC2 and CDX2 were positive in colon tissues, and SOX2 was expressed in tonsil tissues, indicating that all the used antibodies worked well. Scale bars: black bar = 500 μm, white bar = 100 μm.

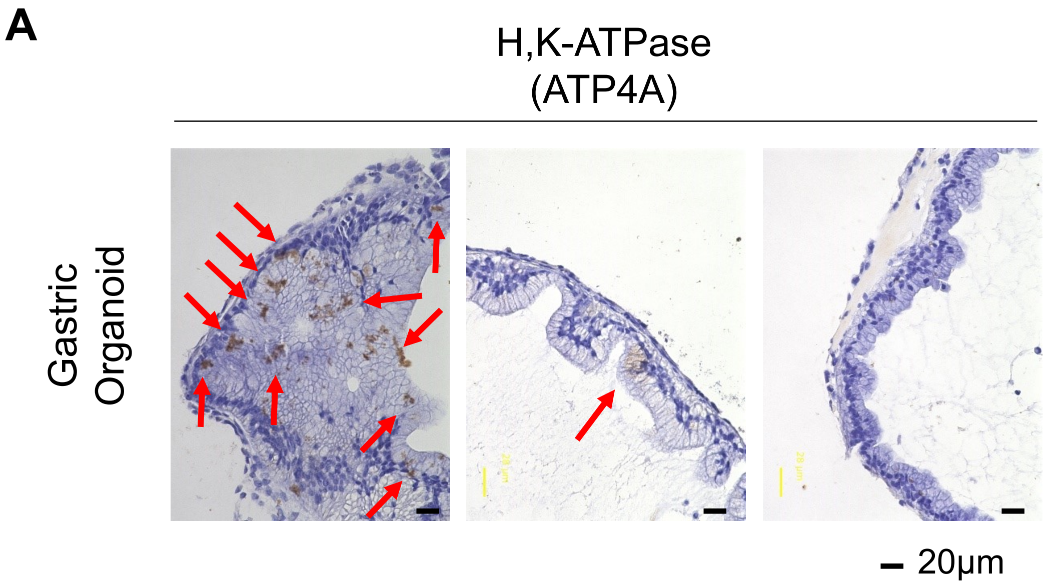

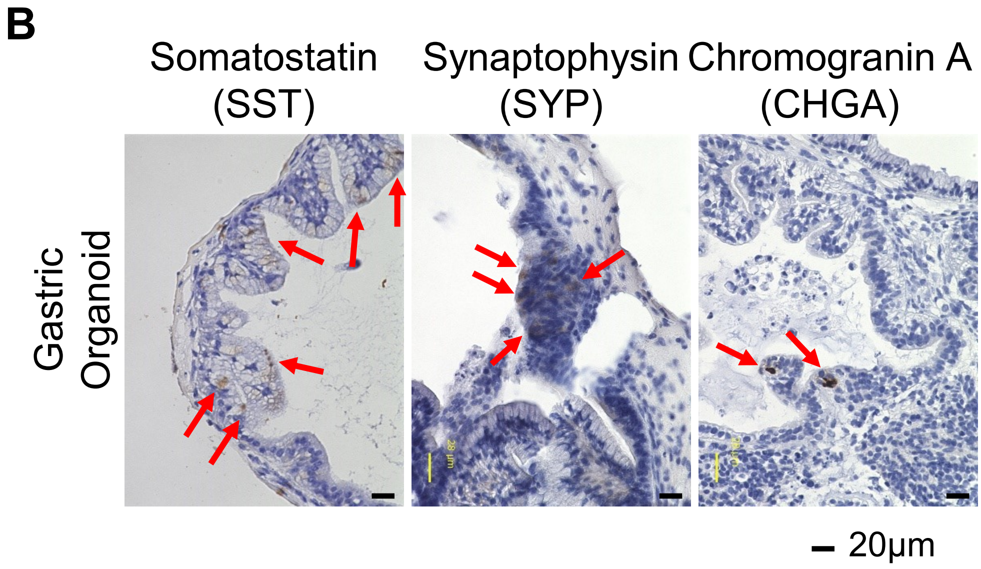

Figure S5. Immunostaining for parietal cell and endocrine cell markers, Related to Figure 3. Immunohistochemical analyses revealed the expression of the parietal cell marker H, K-ATPase (ATP4A) (A) and several endocrine markers (Somatostatin, Synaptophysin and Chromogranin A) (B) in gastric organoids differentiated for ≥40 days. Arrows indicate positive cells. Scale bars, 20 µm.

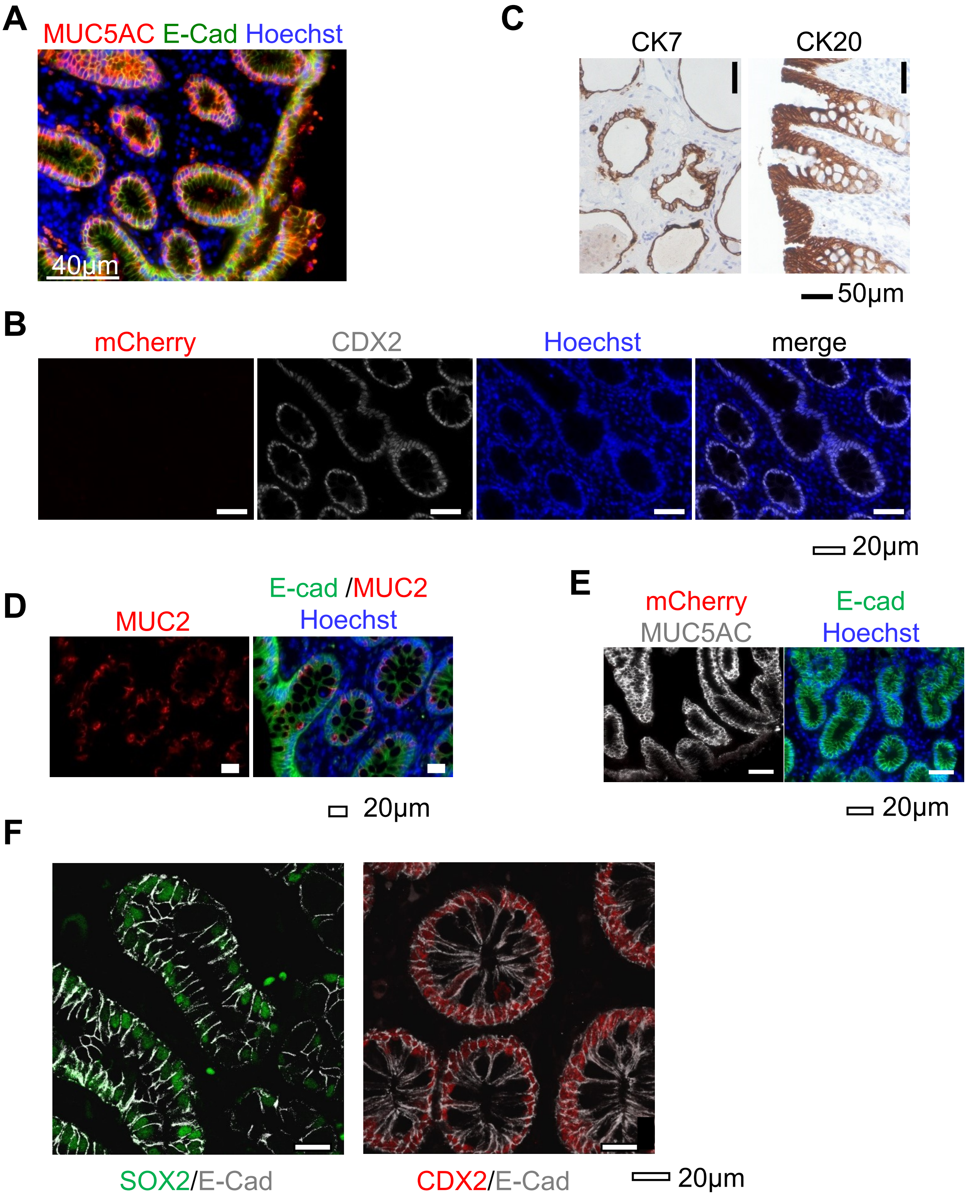

Figure S6. Positive controls for immunohistological analyses of gastro-intestinal marker genes in induced hGOs with or without DOX treatment, Related to Figure 3, 4 and 6. A. Staining of MUC5AC and E-Cadherin in human stomach tissue. Scale bar, 40 μm. **B**. Staining of CDX2 in human colon tissue. Scale bar, 20 μm. **C**. Staining of CK7 in human thyroid tissue and CK20 in human colon tissue. Scale bar, 50 μm. **D**. Staining of MUC2 and E-Cadherin in human colon tissue. Scale bar, 20 μm. **E**. Staining of MUC5AC and E-Cadherin in human stomach tissue. Scale bar, 20 μm. **F**. Staining of SOX2 and E-Cadherin in human stomach tissue (left panel), staining of CDX2 and E-Cadherin in human colon tissue (right panel). Scale bar, 20 μm.

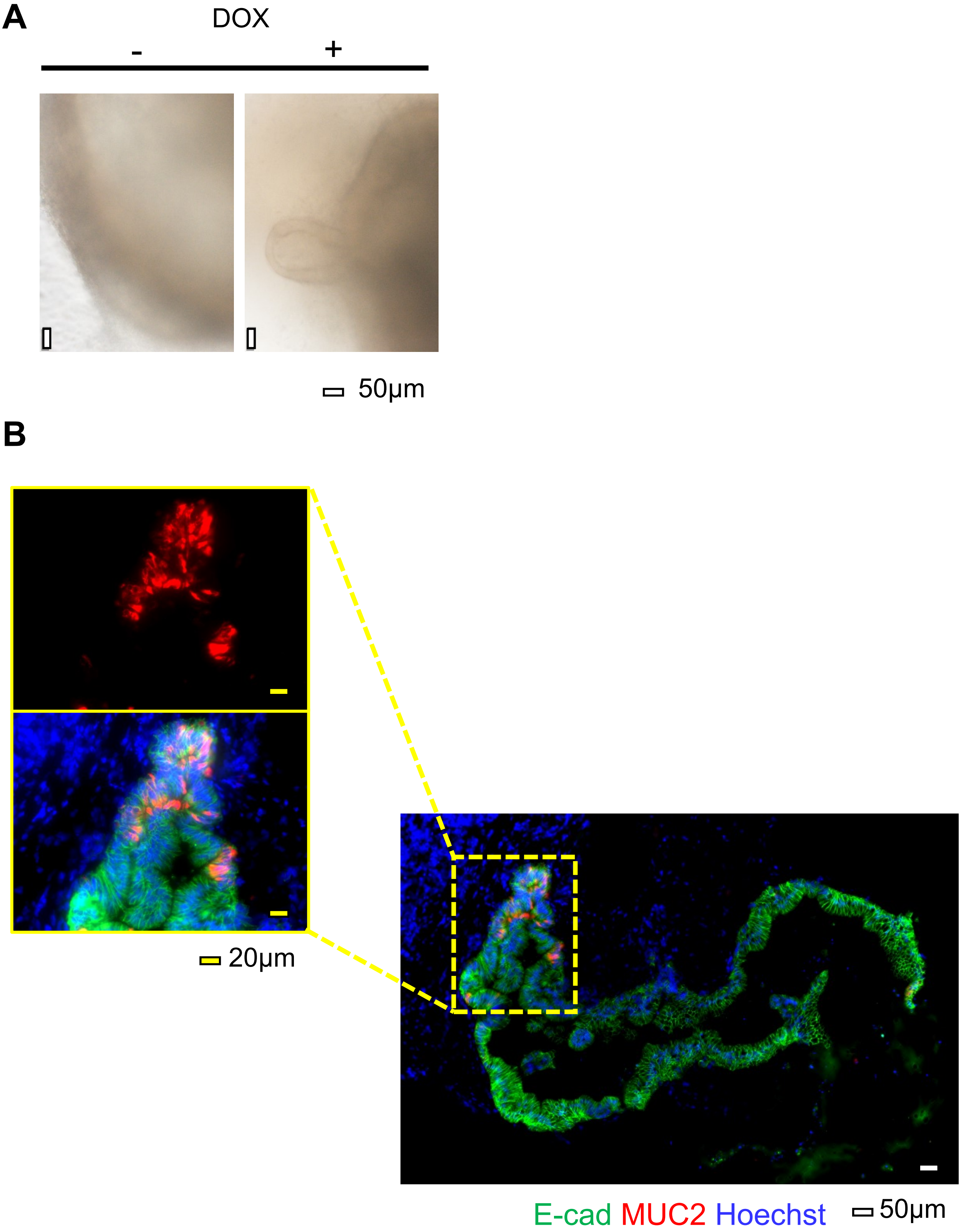

Figure S7. Phenotype alterations in DOX(+) organoids, Related to Figure 4. A. Phase contrast micrographs of the morphology of DOX(-) organoids (left panel) and DOX(+) organoids (right panel). Scale bars, 50 μm. **B.** Immunofluorescence analyses of E-Cadherin and MUC2 expression in the gastric organoids derived from CDX2-iPSC at Day 44 with DOX treatment for 7 days. The crypt-like domain, which protrudes outside the organoid, was boxed in the area of the yellow dotted line in the lower right panel. The enlarged view of this domain is shown in the upper left panels. Scale bars, 50 and 20 μm for yellow bar and white bar, respectively.

A

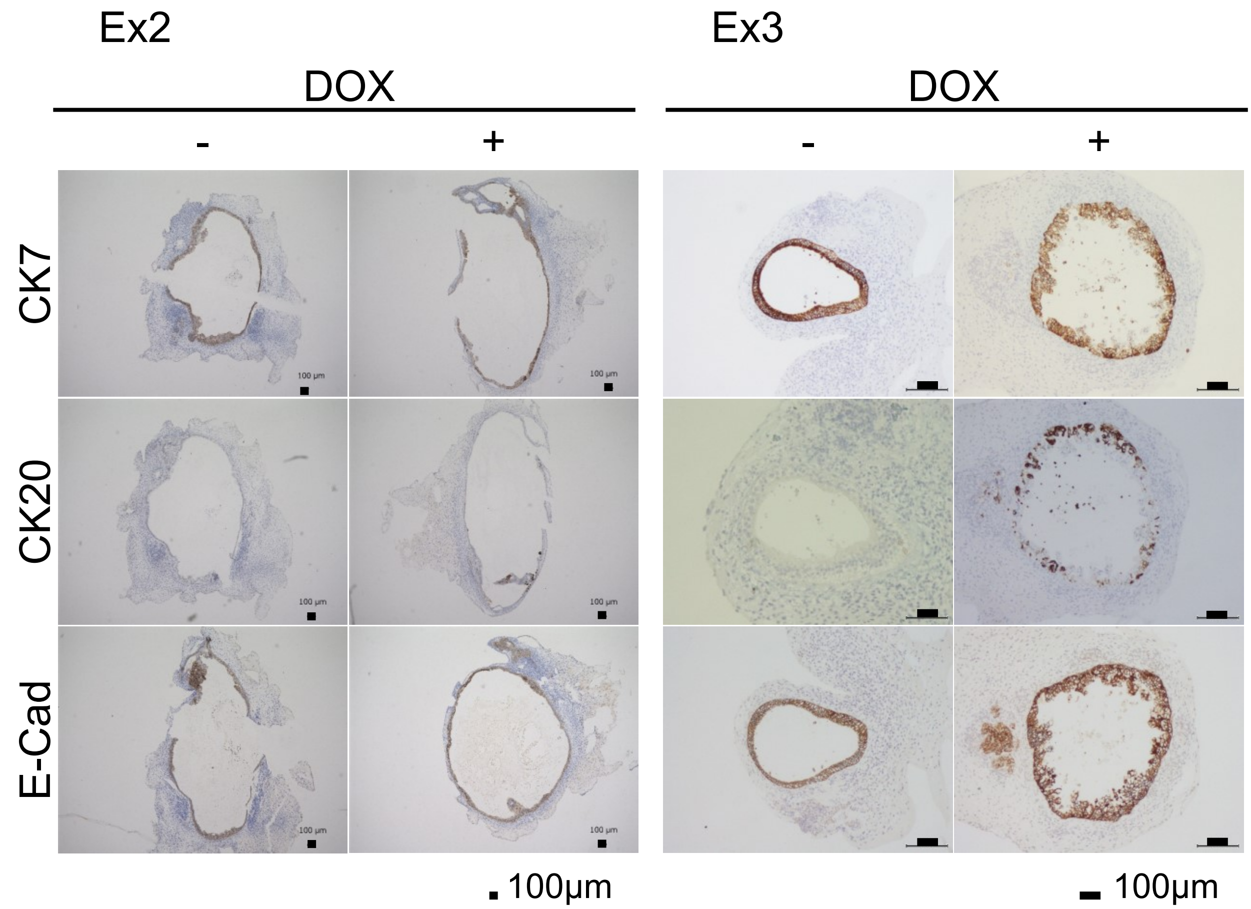

B

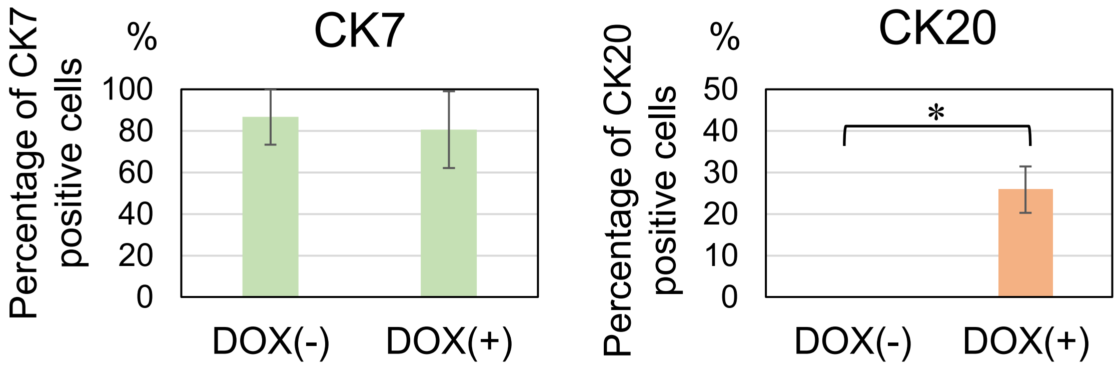

C

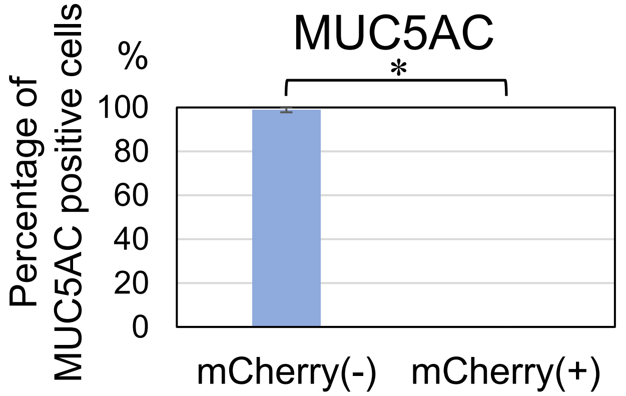

Figure S8. A quantitative immunohistochemical analysis of DOX(-) and DOX(+) organoids, Related to Figure 4. A. Immunohistochemical analyses of CK7, CK20 and E-cadherin in the gastric organoids derived from CDX2-iPSC with (+) or without (-) DOX treatment. Scale bar, 100 μm. The results of two induction experiments different from those in Figure 4B are shown. **B**. The percentages of CK7- and CK20-positive cells in DOX(-) and DOX(+) gastric organoids. The mRNA expression was normalized to GAPDH. Data are represented as mean +/- SEM of three independent induction experiments. *, $p<0.05$. **C**. The percentages of MUC5AC-positive cells in mCherry (-)/E-Cad(+) cells and mCherry (+)/E-Cad (+) cells. The mRNA expression was normalized to GAPDH. Data are represented as mean +/- SEM of three independent induction experiments. *, $p<0.05$.

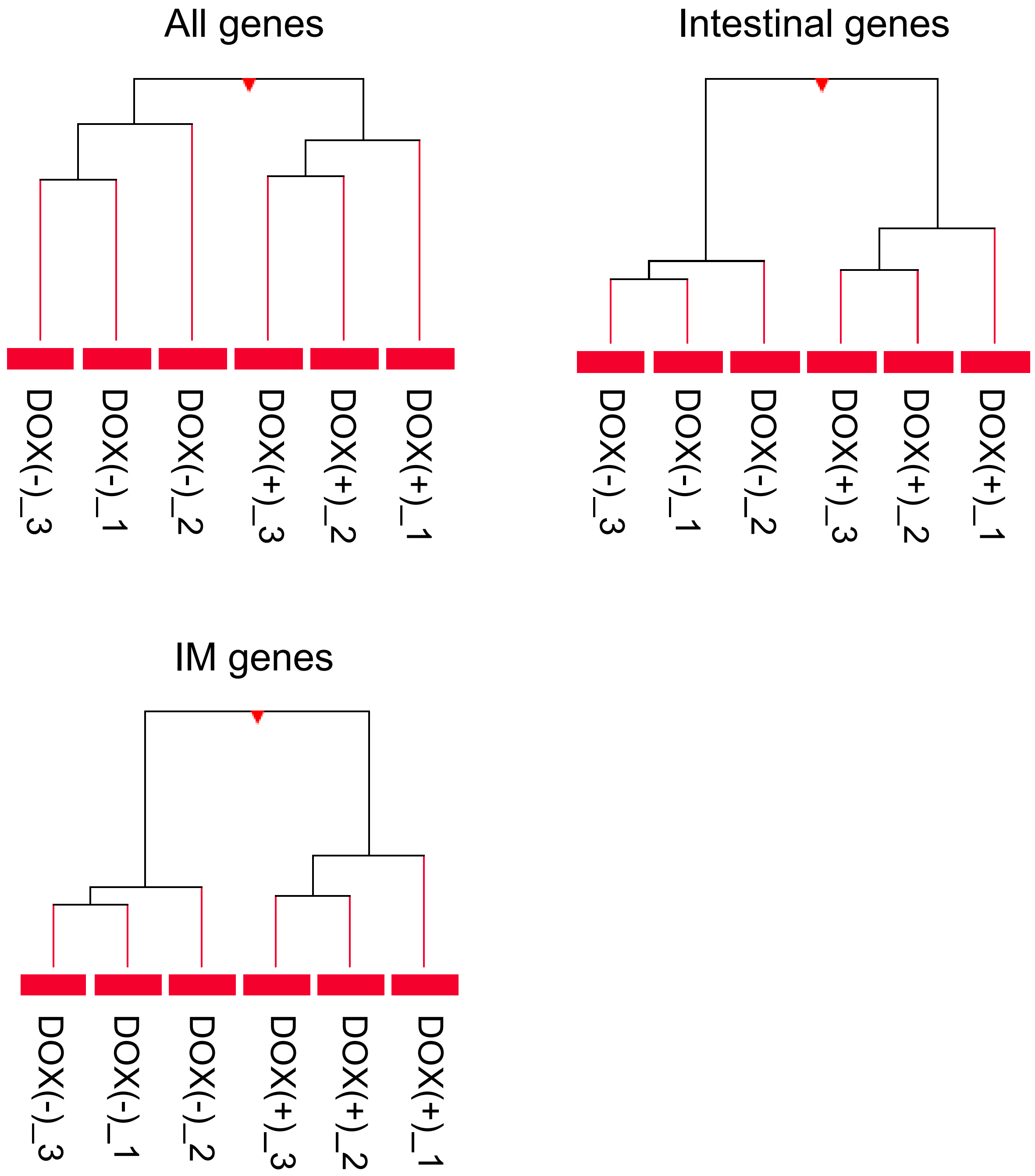

Figure S9. A clustering analysis of NGS, Related to Figure 5. Hierarchical clustering of “All genes”, “Intestinal genes” and “IM genes” are shown. Clustering Algorithm: Hierarchical, Clustering By: Normalized intensity values, Clustering On: Entities and Conditions, Similarity Measure: Euclidean, Linkage Rule: Wards.

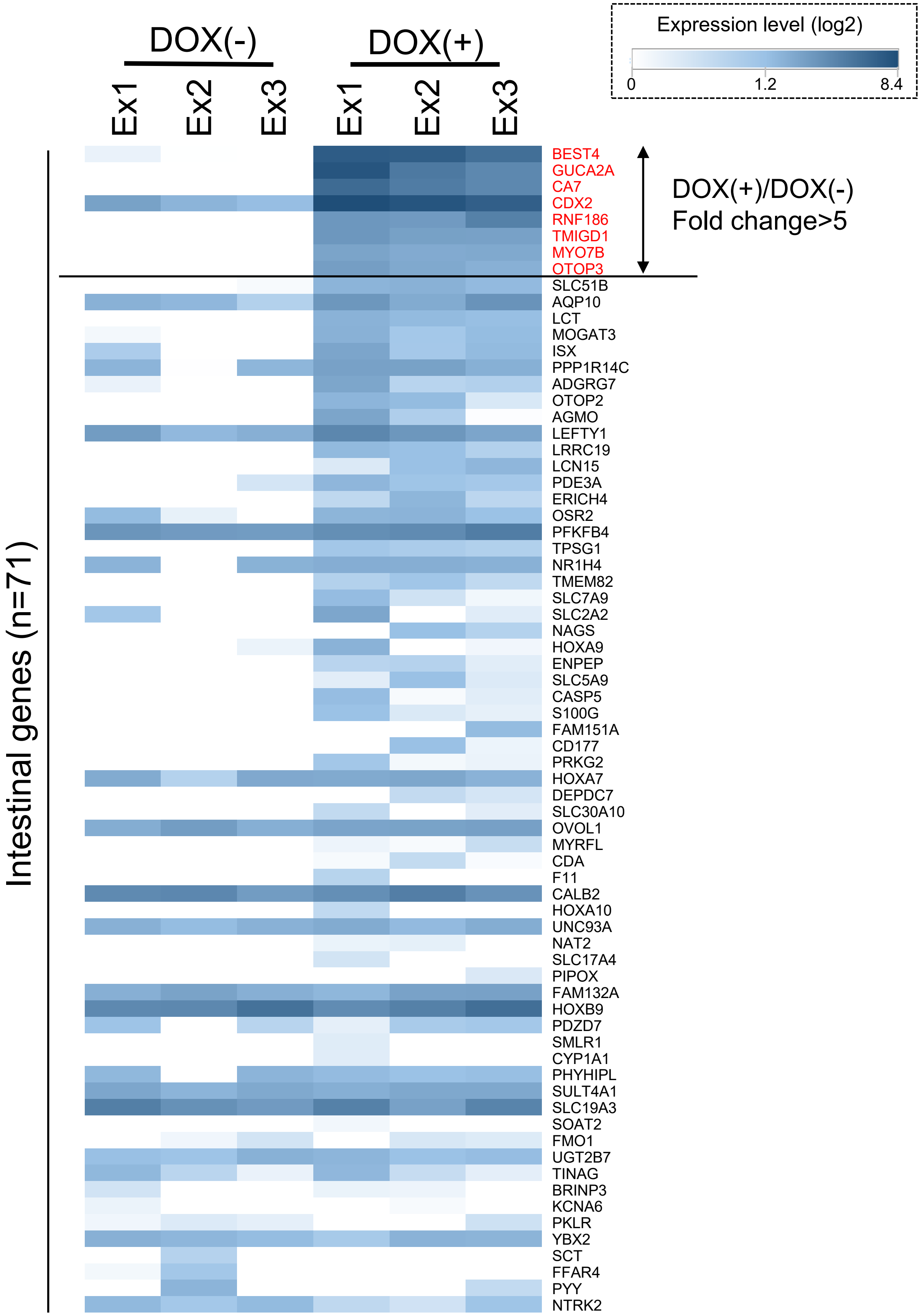

Figure S10. Heatmap showing the intestinal genes on NGS, Related to Figure 5. Heat map for 71 intestinal genes (used in Figure 5) in DOX(-) and DOX(+) organoids (n=3). Of these genes, BEST4, GUCA2A, CA7, RNF186, TMIGD1, MYO7B, OTOP3, and CDX2 were more than 5-fold upregulated on average in DOX(+) samples compared to DOX(-) ones.

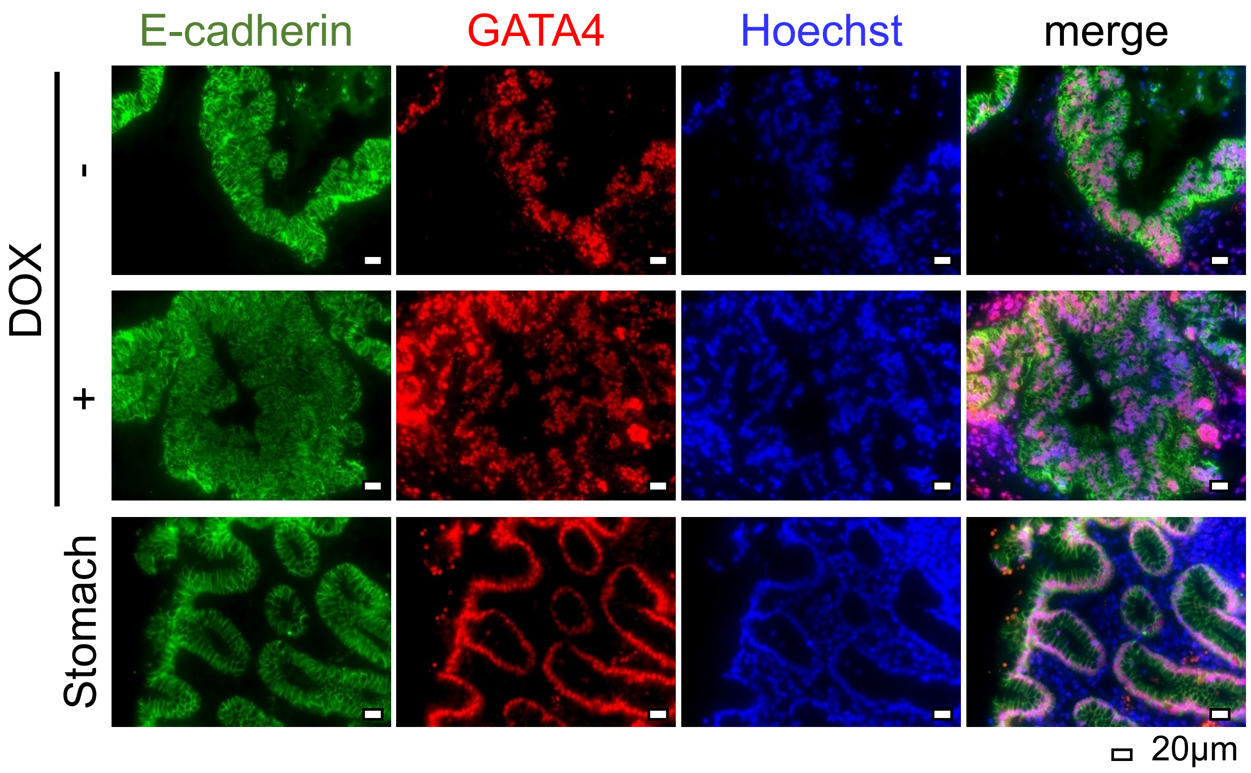

Figure S11. The expression of GATA4 in gastric organoids with or without DOX treatment, Related to Figure 6. Immunofluorescence analyses of E-Cadherin and GATA4 in gastric organoids from CDX2-iPSC at Day 44 without (-) (upper panel) or with (+) (middle panel) DOX treatment for 7 days. We used normal gastric tissue as a positive control for E-Cadherin and GATA4 staining (lower panels). Scale bars, 20 μm.

Table S1. List of primer sequences used for RT-PCR analysis in this study, Related to Figure 1, 2, 3, 4, S1, S2, S8 and STAR Methods.

primer name	sequence
hGAPDH forward	accacagtccatgccatcac
hGAPDH reverse	tccaccaccctgttgctgta
hOCT3/4 forward	ccccagggccccattttggtacc
hOCT3/4 reverse	acctcagtttgaatgcatgggagagc
hSOX2 forward	ttcacatgtcccagcactaccaga
hSOX2 reverse	tcacatgtgtgagaggggcagtgtgc
hNANOG forward	cagccccgattcttccaccagtccc
hNANOG reverse	cggaagattcccagtcgggttcacc
hCDX2 forward	ctggagctggagaaggagtttc
hCDX2 reverse	attttaacctgcctctcagagagc
hPDX1 forward	ctgtgctccagttccacact
hPDX1 reverse	acagcctctacctcggaaca
hMUC5AC forward	ctcagctgttctctggacga
hMUC5AC reverse	gctggatgatcaggctccta
hCK7 forward	aggatgtggatgctgcctac
hCK7 reverse	caccacagatgtgtcggaga
hCK20 forward	ggtcgcgactacagtgcatattaca
hCK20 reverse	cctcagcagccagtttagcattatc
hSOX17 forward	cgctttcatggtgtgggctaaggacg
hSOX17 reverse	tagttggggtggtcctgcatgtgctg
hFOXA2 forward	tgggagcggtgaagatggaagggcac
hFOXA2 reverse	tcatgccagcgcccacgtacgacgac
